# European Society for Pediatric Gastroenterology, Hepatology and Nutrition (ESPGHAN) steatotic liver disease special interest group position paper on screening, diagnosis and investigation of paediatric metabolic dysfunction‐associated steatotic liver disease

**DOI:** 10.1002/jpn3.70490

**Published:** 2026-06-30

**Authors:** Jake P. Mann, Sander Lefere, Maarten Buytaert, Anna Alisi, Cigdem Arikan, Jernej Brecelj, Rachel M. Brown, Irene Degrassi, Giulia Fiore, Emer Fitzpatrick, Sven Francque, Christian A. Hudert, Wojciech Janczyk, Lauren Johansen, Bart G. Koot, Anastasia Konidari, Eberhard Lurz, Claudia Mandato, Maria Mercadal‐Hally, Hadar Moran‐Lev, Antonella Mosca, Giusy Ranucci, Maria Rogalidou, Penelope C. Rose, Piotr Socha, Pietro Vajro, Elvira Verduci, Anita C. E. Vreugdenhil, Natalia Zavhorodnia, Aglaia Zellos, Ruth De Bruyne

**Affiliations:** ^1^ Department of Immunology & Immunotherapy, School of Infection, Inflammation and Immunology, College of Medicine and Health University of Birmingham Birmingham UK; ^2^ Liver Unit (Including Small Bowel Transplantation) Birmingham Women′s and Children′s NHS Foundation Trust Birmingham UK; ^3^ Hepatology Research Unit, Department of Internal Medicine and Pediatrics Ghent University Ghent Belgium; ^4^ Genetic of Complex Phenotype Research Unit Bambino Gesù Children′s Hospital, IRCCS Rome Italy; ^5^ Department of Pediatric Gastroenterology and Organ Transplant Koç University School of Medicine Istanbul Turkey; ^6^ Department of Gastroenterology, Hepatology and Nutrition, University Children′s Hospital Ljubljana, and Department of Pediatrics, Faculty of Medicine University of Ljubljana Ljubljana Slovenia; ^7^ Department of Histopathology Birmingham Children′s Hospital Birmingham UK; ^8^ Department of Health Sciences University of Milan Milan Italy; ^9^ Pediatric Unit Foundation IRCCS Ca′ Granda Ospedale Maggiore Policlinico Milan Italy; ^10^ Department of Paediatric Gastroenterology and Hepatology Children′s Health Ireland Dublin Ireland; ^11^ Department of Gastroenterology and Hepatology Antwerp University Hospital Edegem Belgium; ^12^ InflaMed Centre of Excellence, Laboratory for Experimental Medicine and Paediatrics, Translational Sciences in Inflammation and Immunology, Faculty of Medicine and Health Sciences University of Antwerp Wilrijk Belgium; ^13^ Department of Paediatric Gastroenterology, Nephrology and Metabolic Diseases Charité – Universitätsmedizin Berlin Berlin Germany; ^14^ Department of Gastroenterology, Hepatology, Feeding Disorders and Pediatrics Children′s Memorial Health Institute Warsaw Poland; ^15^ Department of Pediatric Gastroenterology, Emma Children′s Hospital, Amsterdam UMC University of Amsterdam Amsterdam The Netherlands; ^16^ Department of Paediatrics Paidon P&A Kyriakou Children′s Hospital Athens Greece; ^17^ Department of Pediatrics, Dr. von Hauner Children′s Hospital KUM University Hospital LMU Munich Munich Germany; ^18^ Department of Medicine, Surgery and Dentistry University of Salerno Baronissi Italy; ^19^ Pediatric Hepatology and Liver Transplant Unit, Department of Pediatrics Vall d′Hebron Hospital Campus, ERN Rare Liver, ERN TransplantChild Barcelona Spain; ^20^ Department of Pediatric Gastroenterology, Dana‐Dwek Children′s Hospital, Faculty of Medicine Tel Aviv University Tel Aviv Israel; ^21^ Pediatric Department for the Treatment and Study of Abdominal Diseases and Abdominal Transplantation Mediterranean Institute for Transplantation and Advanced Specialized Therapies (IRCCS ISMETT) Palermo Italy; ^22^ Division of Gastroenterology & Hepatology, 1st Pediatrics Department, National and Kapodistrian University of Athens “Agia Sofia” Children′s Hospital Athens Greece; ^23^ Department of Paediatrics and Child Health Stellenbosch University Cape Town South Africa; ^24^ Department of Paediatrics Aalborg University Hospital Aalborg Denmark; ^25^ Centre for Overweight Adolescent and Children′s Healthcare (COACH), Department of Pediatrics, School of Nutrition and Translational Research in Metabolism (NUTRIM), Maastricht University Medical Center+ MosaKids Children′s Hospital Maastricht The Netherlands; ^26^ Department of Pediatric Gastroenterology SI “Institute of Gastroenterology of the National Academy of Medical Sciences” Dnipro Ukraine

**Keywords:** diabetes, elastography, liver biopsy, obesity, steatohepatitis

## Abstract

Metabolic dysfunction‐associated steatotic liver disease (MASLD) is the most common reason for elevated liver enzymes in children in Europe, affecting more than 5% of all children. Since the last iteration of this position paper, there have been substantial advances in our understanding of the disease. After a detailed literature review and thorough discussion, we established consensus recommendations for the diagnosis and assessment of MASLD in children. Alanine aminotransferase (ALT) ≥ 30 IU/L is a suitable screening test for MASLD in children over 10 years of age with obesity (body mass index *z*‐score ≥+2), or in children of any age with additional risk factors. All patients with suspected MASLD should be assessed for alternative or concomitant diagnoses, as well as comorbidities. Patients who are not overweight, under 8 years old, or have any other red flags should be promptly referred for specialist assessment. Liver biopsy remains the gold standard for diagnosis and staging of MASLD, but should be reserved for diagnostic uncertainty, to guide treatment decisions, and risk stratification (e.g., prior to transition to adult care). While there is emerging data for non‐invasive tests (e.g., transient elastography), it is unclear how to routinely implement these investigations in clinical practice. Combined changes of ≥20% in both ALT and gamma‐glutamyl transferase may represent a useful non‐invasive tool for monitoring disease severity over time. Most patients are appropriately investigated and managed by non‐specialists where the focus is on holistic management of obesity and its complications. Future research should focus on how to use non‐invasive tests to risk‐stratify children, in particular, how to identify those with advanced fibrosis.

## Abstract (highlights)

**What is Known:**

- Metabolic dysfunction‐associated steatotic liver disease (MASLD) is the most common reason for persistent elevation of liver enzymes in children.
- Patients with suspected MASLD should be tested for alternative causes of liver disease.
- MASLD is strongly associated with obesity.

**What is New:**

- Screen for MASLD in children over 10 years with obesity (body mass index z‐score ≥+2), or at any age in children with additional risk factors (e.g., insulin resistance).
- Combined changes of ≥20% in both alanine aminotransferase and gamma‐glutamyl transferase are helpful in monitoring the progression or regression of MASLD.
- Transient elastography can help exclude significant liver fibrosis (<5.5 kPa) and identify children at risk of advanced chronic liver disease (>10 kPa).
- Liver biopsy can be used for diagnostic uncertainty, treatment decisions and risk stratification.

## Graphical abstract

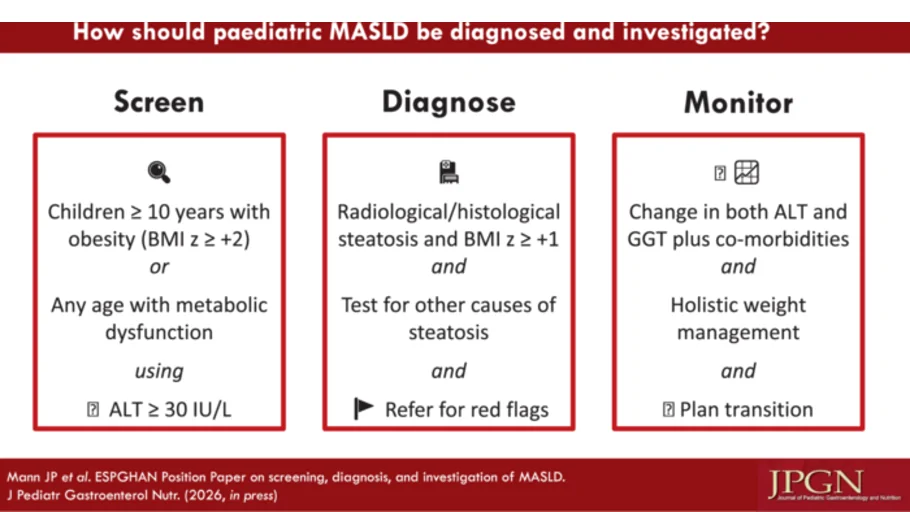

## 1 INTRODUCTION

Metabolic dysfunction‐associated steatotic liver disease (MASLD) is a chronic condition characterised by the accumulation of excess lipids in hepatocytes.^1^ It is the most common cause of persistently elevated liver enzymes in children.^2^ MASLD is the second most common indication for liver transplantation in adults in the United States^3^ and is becoming a leading indication in Europe.^4^ MASLD is driven by a combination of insulin resistance (typically secondary to excess adiposity),^5^ genetics^6^ and additional acquired factors (e.g., obstructive sleep apnoea).^7^ Recent data have demonstrated that paediatric onset MASLD confers a 40‐fold increase in standardised mortality ratio in young adulthood,^8^ as well as a 20% risk of incident type 2 diabetes within 5 years.^9^

Since the last European Society for Paediatric Gastroenterology Hepatology and Nutrition (ESPGHAN) position paper on this topic,^10^ nomenclature has shifted from non‐alcoholic fatty liver disease (NAFLD), through metabolic dysfunction‐associated fatty liver disease (MAFLD),^11^ to MASLD. There have also been substantial developments in technologies used for the assessment of MASLD, such that non‐invasive algorithms can effectively risk stratify adults with MASLD.^12^ Concurrently, greater familiarity with the disorder has also led to less frequent use of liver biopsy among both adult and paediatric hepatologists, though marked heterogeneity in practice remains.^13^

Research on children affected with MASLD over the last 13 years has identified: clinical and biochemical factors associated with more severe disease^14^, ^15^; how genetic variation influences histological phenotypes^16^; and blood or imaging biomarkers of fibro‐inflammation.^17^, ^18^, ^19^ These advances and greater clinical experience of the condition form the basis for this updated position paper.

In this updated position paper, we seek to provide evidence‐based recommendations for diagnosis and assessment of MASLD in children. Treatment and management^20^, ^21^, ^22^ are beyond the scope of this paper and will be incorporated into a subsequent position paper.

## 2 METHODS

### 2.1 Scope

This position paper was initiated in 2024 by the ESPGHAN Special Interest Group on MASLD (SIG‐MASLD). Committee discussion and literature review were used to identify questions and sub‐questions to address screening and diagnosis of paediatric MASLD (Table 1), in conjunction with the ESPGHAN Hepatology Committee. The committee determined that the focus of this position paper would be on diagnosis and assessment of MASLD in children.

**Table 1:** Identified questions to address as part of the position paper.

Questions addressed	Topics/sub‐tasks
How effective is each modality at detecting (a) the presence of‐, and (b) change in each of steatosis, inflammation and fibrosis? How suitable is each modality for screening? And identification of high‐risk/severe cases? And monitoring of progression?	Common liver enzymes (ALT, AST, GGT, etc)
	Histology
	Magnetic resonance imaging
	Transient elastography (CAP & shearwave elastography)
	Ultrasound
	Composite non‐invasive scores (e.g., PNFI), and ‘novel′ biomarkers (e.g., ELF test)
Who should (if anyone) be screened for paediatric MASLD?	Who is at the highest risk? Who should be screened (considering the WHO pre‐requisites for screening)? How can clinical history/examination help? Is there evidence for an optimal screening interval?
Which alternative diagnoses should be ruled out in a patient with (suspected & confirmed) paediatric MASLD? And how?	For each alternative diagnosis: what is the minimum investigation to be performed? When is biopsy indicated for diagnostic purposes? When to refer to alternative specialty (e.g., endocrinology, inherited metabolic disease)?
Which comorbidities should be tested for? (e.g., hypertension, diabetes)	For each co‐morbidity: what is the minimum investigation to be performed? When to refer to an alternative speciality (e.g., endocrinology)?
What is the role of genetic testing in current clinical practice?	Including use of targeted SNPs (e.g., PNPLA3) and exome/genome analysis for diagnostic uncertainty.
How should follow‐up be conducted?	How long to follow‐up? When should be done by gastro‐hepatologist, paediatrician, family, doctor, or other specialty? How often to use each diagnostic test in follow‐up?
When to refer to a paediatric gastroenterologist/hepatologist?	Which part of the work‐up can be done by a family doctor or a first‐line paediatrician? Which are the criteria to refer to a paediatrician with special interest in gastroenterology ‐ hepatology (tertiary care level)?

*Note*: Committee discussion and literature review were used to identify questions to address, in conjunction with the ESPGHAN Executive Committee and Hepatology Committee.

Abbreviations: ALT, alanine aminotransferase; AST, aspartate aminotransferase; CAP, controlled attenuation parameter; ELF, enhanced liver fibrosis test; GGT, Gamma‐glutamyl transferase; MASLD, Metabolic‐dysfunction associated steatotic liver disease; PNFI, paediatric NAFLD fibrosis score; SNPs, single nucleotide polymorphisms.

### 2.2 Search strategy and formulation of recommendations

Medline (PubMed), EMBASE, Google Scholar, Cochrane Library and Scopus were searched up to April 11, 2025 using: (‘Fatty liver (disease)’ OR “NAFLD’ OR ‘non‐alcoholic‐fatty‐liver‐disease’ OR ‘NASH’ OR ‘liver steatosis’, ‘steatotic liver disease’ OR ‘obesity‐related liver disease’ OR ‘MASLD’ OR ‘metabolic dysfunction‐associated‐liver‐disease’ OR ‘steatohepatitis’) AND (‘children’ OR ‘pediatrics’ OR ‘adolescent’). Search identified 3714 articles, of which 904 were duplicates. 2810 abstracts were reviewed and 221 articles were included as the evidence base for the position paper. Included articles were reviewed by at least two authors. Evidence was used to formulate recommendations across the key questions.

### 2.3 Grading of evidence

The levels of evidence (LoE) for all recommendations were derived according to the Oxford Centre for Evidence‐based Medicine system.^23^ Where the committee determined that recommendations were required despite a lack of suitable evidence, a recommendation was developed based on expert opinion and consensus.

### 2.4 Consensus

The draft statements and recommendations of the position paper committee underwent at least two rounds of discussion with the SIG‐MASLD. All participating members were invited to vote on agreement with statements and recommendations via Qualtrics using a four‐point scale (‘agree’; ‘somewhat agree’, ‘somewhat disagree’, ‘disagree’). For inclusion in the final position paper, consensus had to be reached which was defined as at least 80% of the members responding with either ‘agree’ or ‘somewhat agree’, ≥95% agreement was classified as ‘strong consensus′. Consensus was obtained for all statements and recommendations after the first voting round. However, based on the level of agreement and the feedback provided during the first voting round, 15 statements and recommendations were reformulated and underwent a second voting round. The results of the first and the second voting rounds are summarised and visualised in Table S1a,b and Figure S1. Within the manuscript, the percentage of agreement (which is the sum of the votes ‘somewhat agree’ and ‘totally agree’) is given with each recommendation (File S1). The final draft of the manuscript was sent to all contributing authors for approval in April 2026.

### 2.5 Definitions

In 2023, an international multi‐society consensus renamed and redefined NAFLD as MASLD.^24^ The aim of this was to reduce stigma, move towards a diagnosis of inclusion, and allow for concurrent diagnoses. MASLD is defined as the presence of hepatic steatosis with metabolic dysfunction. ‘Metabolic dysfunction′ refers to components of the metabolic syndrome (termed cardiometabolic criteria), the features associated with insulin resistance, typically elicited by being overweight (body mass index [BMI] *z*‐score ≥1 and <+2) or affected with obesity (BMI *z*‐score ≥ +2).^25^ Metabolic dysfunction‐associated steatohepatitis (MASH) is a histological diagnosis that requires demonstration of hepatic steatosis with inflammation and/or fibrosis, in conjunction with metabolic dysfunction.

ESPGHAN and other paediatric gastro‐hepatology societies have endorsed this new terminology, with some caveats.^1^ In particular, diagnosing MASLD in non‐overweight individuals should be reserved for specialists. It has been noted that MASLD is not a lay‐friendly term and is particularly challenging to explain to young people with MASLD.^26^, ^27^ However, there is also a stigma associated with using the term ‘fatty′^28^.

#### 2.5.1 Recommendations

1. In scientific research and clinical practice, MASLD and MASH are to be used as terminology for NAFLD and non‐alcoholic steatohepatitis, respectively (LoE 3; 100%; strong consensus).

## 3 SCREENING FOR PAEDIATRIC MASLD

### 3.1 Should we screen?

MASLD is a global public health concern^29^ and the disease burden is continuing to rise across Europe.^30^, ^31^ Efficient paediatric screening programmes can lead to targeting of lifestyle modifications and closer follow‐up with a reduction in severity and an impact on long‐term morbidity and mortality.^32^, ^33^ At the same time, during the growing obesity epidemic, overdiagnosis should be avoided as this can lead to healthcare strain and increase patient anxiety. It has been argued that a diagnosis of MASLD should carry clinically significant prognostic information, rather than simply being a label.^34^

The natural history of paediatric MASLD has been described in long‐term follow‐up studies both at a population level and from specialist cohorts. When 1096 children from the ultrasound (US) with MASLD were followed‐up for a mean of 8.5 years, 3.4% died (almost half liver‐related deaths) at a standardised mortality rate of 40‐times increased compared to age‐/sex‐matched controls.^8^ Similarly, in a study of 170,000 Swedish children, childhood obesity was associated with a 2.2 times increased risk of major adverse liver outcomes over 8.5 years follow‐up.^35^ Data from shorter‐term retrospective studies using liver biopsy or magnetic resonance elastography (MRE) show, over 2–3 years, roughly one‐third of children have disease progression, 20% have disease regression, and 50% remain stable.^36^, ^37^, ^38^, ^39^ Therefore, while clinical end‐stage liver disease due to MASLD does not tend to develop in childhood, follow‐up studies demonstrate that MASLD is a chronic, progressive disorder associated with increased mortality in young adulthood.^8^

Considering the benefits and harms of an effective screening programme (e.g., the acceptability of the test, presence of a latent stage, availability of suitable facilities for diagnosis and treatment),^40^, ^41^ paediatric obesity guidelines and nearly all paediatric MASLD guidelines recommend screening for paediatric MASLD.^42^

### 3.2 Who to screen?

**Figure 1:**
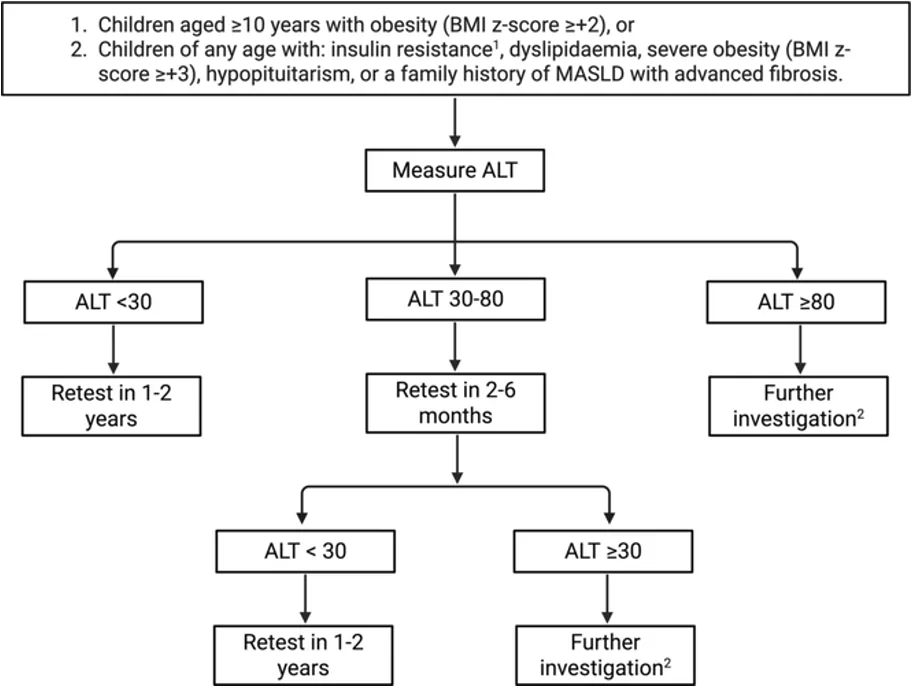
Screening for MASLD in children. Children over 10 years old with obesity (BMI *z*‐score ≥+2) and those of any age with additional risk factors should have ALT measured. Children who are overweight and not obese (BMI *z*‐score ≥1 and <2) should not be screened unless there are additional signs of insulin resistance, dyslipidaemia, or risk factors for MASLD. If ALT is under 30 IU/L, repeat screening in 1–2 years. If ALT is over 80 IU/L then perform clinical assessment and investigations. If ALT is 30–80 IU/L, and there is no concern for acute liver disease, repeat testing in 2–6 months. ^1^Signs of insulin resistance include: type 2 diabetes, pre‐diabetes, biochemical insulin resistance and acanthosis nigricans. ^2^Further investigation does not always require referral. ALT, alanine aminotransferase; BMI, body mass index; MASLD, metabolic dysfunction‐associated steatotic liver disease.

The most common risk factor for MASLD is obesity, with up to half of all adolescents affected by obesity having some evidence of MASLD.^43^ Most of the screening recommendations are based on expert opinion and advise screening of all children living with obesity (BMI *z*‐score ≥+2) and those who are overweight (BMI *z*‐score ≥1 and <+2) with additional risk factors (such as central adiposity, insulin resistance, prediabetes, diabetes, dyslipidaemia, obstructive sleep apnoea (OSA), or a family history of MASLD^10^, ^44^, ^45^, ^46^, ^47^). Here, we recommend screening children with obesity (BMI *z*‐score ≥+2), as severity of metabolic dysfunction (and degree of obesity) correlates with severity of MASLD.^48^ Children who are overweight and not obese (BMI *z*‐score ≥1 and <+2) should not be screened unless they have additional risk factors (Figure 1).^10^, ^44^, ^45^, ^46^, ^47^

No evidence is available regarding the most appropriate age to start screening. However, it is reasonable to start screening around the age of 10 years, as advanced liver disease due to MASLD is rare below that age, and scientific data have almost exclusively been collected in cohorts of children aged 10 years or older. Screening at a younger age risks overdiagnosis in children without signs of end‐organ metabolic dysfunction.^49^, ^50^ Given the strong association between insulin resistance and MASLD severity, earlier screening may be considered in younger patients as well as those with a family history of MASLD and advanced fibrosis (Figure 1).^5^, ^51^ In addition, acquired hypopituitarism (and hypothalamic insufficiency) has been associated with rapidly progressive MASLD.^37^

This is a pragmatic approach and factors that influence this include: acceptability of blood tests for children,^52^, ^53^ uncertainty over the natural history of MASLD in this age group,^37^, ^49^ and the lack of available pharmacotherapies. In addition, we recognise that there is uncertainty around defining weight cut‐offs, as validation of growth charts in children with obesity has predominantly been performed in non‐European cohorts.^54^

In screening for MASLD, we recommend that signs of metabolic dysfunction primarily focus on metrics of insulin resistance and dyslipidaemia. These characteristics are consistently associated with the presence and severity of MASLD in children.^14^, ^48^ While hypertension is a further common finding in patients with MASLD (and included as part of diagnostic cardiometabolic criteria), isolated hypertension (in the absence of obesity or insulin resistance) is more typical of renal or cardiac disease.^55^ Children with both hypertension and dyslipidaemia would warrant screening for MASLD.^56^

#### 3.2.1 Recommendations

2. MASLD screening should start at age 10 in children with obesity (BMI *z*‐score ≥+2), or earlier in children with evidence of insulin resistance, dyslipidaemia, severe obesity (BMI *z*‐score ≥ +3), positive family history for MASLD with advanced fibrosis, or hypopituitarism. (LoE 4; 96%; strong consensus).

### 3.3 How to screen?

Although there is no uniformity on what screening tool is recommended, ALT is the modality with the highest consensus. The optimal threshold is, however, still unclear and varies between guidelines.^10^, ^44^, ^45^, ^46^, ^47^

ALT is a nonspecific marker that can arise from the liver, heart and muscle.^57^ As the most sensitive and liver‐specific liver enzyme, ALT remains widely used to indicate liver injury, due to its ease, cost‐effectiveness and limited invasiveness. Defining an optimal cut‐off for ALT in the detection of steatosis is crucial. Despite its limitations, up to this point, no other single tool has proven to be superior to ALT in the screening of paediatric MASLD.

The American Association for the Study of Liver Disease (AASLD) has recently endorsed use of any degree of ALT elevation above sex‐specific National Health and Nutrition Examination Survey (NHANES)‐based upper limit of normal (ULN).^58^ They recommended transaminases as a single‐test screening strategy for suspected MASLD, overtaking previous ESPGHAN^10^ recommendation of ALT and US.

As a screening test, in children with obesity, ALT can have high specificity (88%–92%)^59^ for the presence of steatosis, though this comes with low sensitivity (48%–52%). The sensitivity is influenced by the cut‐off values used. The most frequently used ULN were derived from the NHANES population (in teenagers over 12 years of age). The 95th percentile levels for ALT in healthy weight, metabolically normal, liver disease‐free patients were 22 U/L for girls and 26 U/L for boys. The 2010 Screening ALT for Elevation in Today′s Youth (SAFETY) study found that a fixed ALT threshold of twice the ULN was optimal for diagnosing magnetic resonance imaging (MRI)‐verified MASLD in North‐American teenagers who were overweight or had obesity.^60^ Using the SAFETY ULN as cut‐off has higher sensitivity (74%–81%)^61^, ^62^ compared to 2x‐ULN. Applying the SAFETY ULN to the US population gives 40% of adolescents with obesity have ALT values above ULN and 6.6% have ALT >2x ULN.^63^ European data suggested higher ALT ULN (24–29 U/L for girls and 29–32 U/L for boys) in children aged 6–8 years.^64^ Another large European study found 97th percentile cut‐offs also to be higher than those in the NHANES study: 24–32 U/L in girls and 30–38 U/L in boys, with the peak during early puberty.^65^ Hence, no universal reference for normal ALT levels in paediatric steatosis accounts for the complexities of age, sex, pubertal stage and ethnicity, underlining the need for age‐, sex‐ and population‐specific reference ranges.^66^

In contrast to US, liver enzymes do provide evidence for risk stratification. While advanced fibrosis is described in children with normal liver enzymes, and up to 12% of children with normal liver enzymes have fibrosis,^67^ the degree of elevation of ALT is associated with the risk of steatohepatitis and fibrosis stage.^17^, ^38^, ^68^ Therefore, the identification of radiological steatosis with near‐normal liver enzymes (ALT <2x ULN) is less likely to be clinically significant.

For guidelines to be effectively implemented, particularly for non‐specialists, they must be simple.^69^, ^70^ Therefore, given the uncertainty over precise levels, here we suggest ALT 30 IU/L as a uniform screening cut‐off. This will help facilitate implementation in primary care. An alternative approach could have been to use a percentile‐based threshold of ALT, however, we do not recommend this because it would require highly specialist reporting of age‐, sex‐, ethnicity‐ and pubertal stage‐adjusted references ranges, for which there is insufficient data. There is also a relative lack of published data from Eastern and Southern Europe.

We recommend a higher cut‐off of 80 IU/L to instigate mandatory immediate further investigation, as this may be a sign of liver disease of alternative aetiology needing treatment.^57^, ^71^, ^72^ When ALT is between 30 and 80 IU/L on initial testing, and there is no concern for acute liver disease,^58^ we recommend repeating testing in 2–6 months (depending on the clinical situation) to avoid over‐investigation, for example, those who have a transient rise in transaminases following a viral illness. A persistently raised ALT ≥ 30 IU/L should be investigated (Figure 1).

The additional value of US as a screening tool, either alone or in combination with ALT, has also been evaluated. The use of ALT and US in a stepwise screening protocol was evaluated in one European study in 99 children with severe obesity compared to MRI‐proton density fat fraction (MRI‐PDFF) as the reference diagnostic modality.^62^ ALT and US were found to have sensitivities of 44% and 51%, respectively, and specificities of 89% and 80% for the presence of steatosis on MRI. The sequential use of ALT and US provided no added benefit. A USA‐based study compared the NASPGHAN (ALT>2x ULN, i.e., >52 IU/L for boys and >44 IU/L for girls) and the ESPGHAN (ALT > 45 IU/L and/or fatty liver on US) screening strategies. ESPGHAN guidelines resulted in more MASLD diagnoses (58% vs*.* 26%).^73^ Importantly, children with ‘fatty liver infiltration’ on US and low ALT were less likely to have metabolic syndrome, insulin resistance or hypertriglyceridaemia,^73^ indicating that the combined strategy here potentially over‐diagnosed children with a low risk profile for advanced disease.

#### 3.3.1 Recommendations

3. In children eligible for screening (see recommendation 2), screening for MASLD should be performed using ALT ≥ 30 IU/L. (LoE 4; 100%; strong consensus)

## 4 DIAGNOSIS AND ASSESSMENT OF MASLD

### 4.1 Diagnosis of MASLD

MASLD can co‐exist with other diseases, and hence, diagnosis includes investigation for other causes of liver disease.^1^, ^24^, ^74^ The majority of patients are typically referred following screening or present with nonspecific abdominal pain.^75^ Presentation with end‐stage liver disease or clinically significant portal hypertension would typically suggest a diagnosis other than MASLD.^71^

Clinical assessment should consist of a comprehensive medical history taking (including family history, medication use, lifestyle habits and mental health) and an extensive physical examination (anthropometric measures, stigmata of chronic liver disease, dysmorphic signs and signs of insulin resistance) (Table S2).

A discussion of all potential alternative diagnoses is beyond the scope of this position paper. Overall, the risk of alternative, secondary, or concomitant diagnoses is as low as 1%–2% in patients with US‐detected steatosis,^76^, ^77^ but up to 40% in patients undergoing liver biopsy for possible MASLD in specialist centres.^78^ Therefore, investigation should be pragmatic in all and more extensive in patients at higher risk of alternative diagnoses (e.g., markedly raised liver enzymes, not overweight children (BMI *z*‐score <+1), younger age^79^).

**Figure 2:**
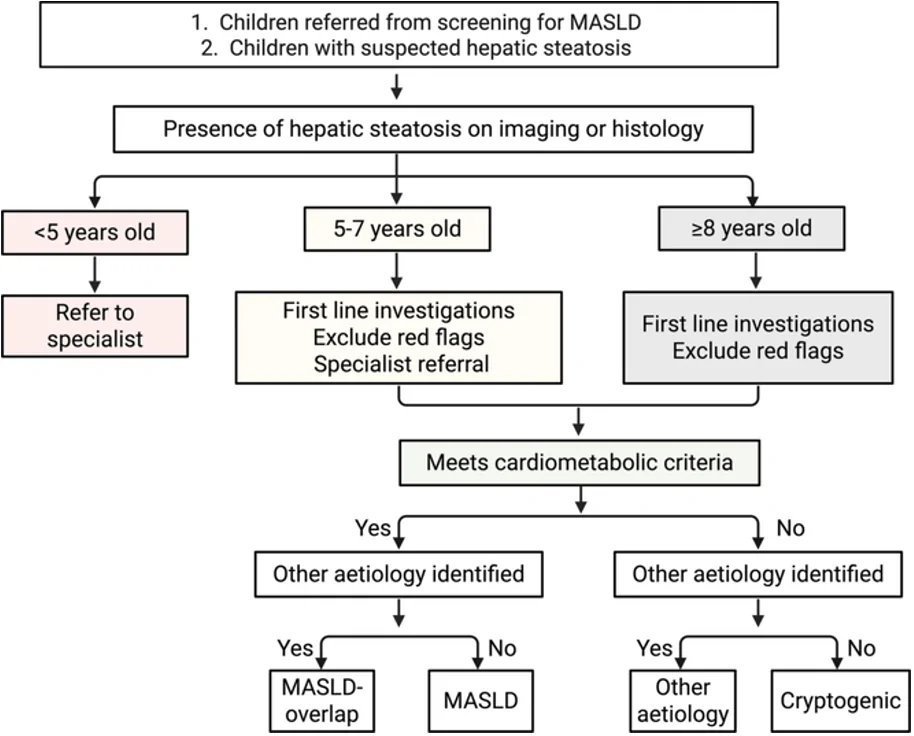
Diagnosis of MASLD in children. First, demonstrate the presence of hepatic steatosis. MASLD is rarely diagnosed under 5 years old. All patients under 8 years old with steatosis should be referred for specialist advice. For children over 8 years old, look for red flags (Table 3) and perform first‐line investigations (Table 2). Then, assess whether they meet any cardiometabolic criteria (Table 4) to identify metabolic dysfunction in order to diagnose MASLD with or without additional diagnoses. Persistently raised liver enzymes and steatosis despite loss of ≥5% total body weight may be a further criterion for suspecting an additional diagnosis causing liver steatosis. MASLD, metabolic dysfunction‐associated steatotic liver disease.

For most patients with suspected MASLD (≥8 years of age and who are overweight (BMI *z*‐score ≥+1), and in the absence of red flags, it is sufficient to test for coeliac disease, Wilson′s disease, autoimmune hepatitis, alpha‐1 antitrypsin deficiency, hypothyroidism and chronic viral hepatitis B or C infection (Table 2). These all have specific therapies and effective screening tests. These tests should be performed in addition to assessing for red flags and considering specialist referral (Figure 2). Prompt investigation is important to avoid missing a diagnosis with a time‐critical therapeutic intervention. The most likely false‐positive test is raised autoantibodies and/or immunoglobulin G,^80^ which will prompt referral to a specialist paediatric gastro‐hepatologist for further assessment. It should be noted that when there is suspicion of autoimmune hepatitis (e.g., raised immunoglobulin G, or patients with red flags, Table 3), comprehensive assessment should also include testing for anti‐LKM1 (liver‐kidney‐microsomal 1), anti‐LC1 (liver cytosol 1), anti‐SLA (soluble liver antigen) antibodies^83^ in addition to the tests in Table 2.

**Table 2:** First‐line investigations in all children with suspected MASLD.

Investigations	Rationale
Abdominal ultrasound	Demonstrate increased echogenicity, evidence of portal hypertension (such as increased spleen size, presence of varices), or other structural abnormalities
ALT, AST, GGT, total and direct bilirubin, albumin, prothrombin time/INR	Baseline assessment of MASLD and liver function
Immunoglobulins, ANA, anti‐SMA	Elevated in autoimmune hepatitis
Caeruloplasmin	Low in Wilson′s disease
Creatine kinase	Elevated in myopathies and mitochondrial disorders
Thyroid function tests	Abnormal in hyper‐/hypo‐thyroidism
Serum A1AT level	Low in A1AT deficiency
IgA anti‐TTG (including total serum IgA)	Raised in Coeliac disease
HBsAg, anti‐HCV	Positive in chronic viral hepatitis

*Note*: These should be performed to identify alternative or additional diagnoses for steatosis and/or raised liver enzymes.

Abbreviations: A1AT, alpha‐1‐antitrypsin; ALT, alanine aminotransaminase; ANA, antinuclear antibody; anti‐SMA, anti‐smooth muscle antibody; AST, aspartate aminotransaminase; GGT, gamma‐glutamyl transferase; HBsAg, hepatitis B surface antigen; HCV, anti‐hepatitis C antibody; IgA, immunoglobulin A; INR, international normalised ratio; MASLD, metabolic dysfunction‐associated steatotic liver disease; TTG, tissue transglutaminase.

**Table 3:** Red flags for detailed investigation.

Red flag	Rationale
Under 8 years old	MASLD rare
ALT ≥ 150 IU/L	Suggestive of liver injury due to alternative aetiology (e.g., Wilson′s disease or autoimmune hepatitis) or more severe MASLD/MASH
GGT ≥ 60 IU/L	Suggestive of biliary injury or more severe MASLD/MASH
Absence of overweight or obesity	Reduces the likelihood of MASLD
Splenomegaly or low platelets	Suggestive of portal hypertension (rare in paediatric MASLD) or storage disorders
Signs of chronic liver disease or portal hypertension	Rare in paediatric MASLD
Signs of synthetic hepatic dysfunction or jaundice	Rare in paediatric MASLD
Growth failure	Suggestive of alternative aetiology (e.g., type 1 diabetes mellitus)
Developmental delay or deteriorating academic performance	Suggestive of alternative aetiology (e.g., mitochondrial disorders)
Syndromic features	Suggestive of alternative aetiology (e.g., Alström syndrome)
Hypopituitarism	Associated with severe MASLD^81^, ^82^

*Note*: The presence of any of these in children being assessed for MASLD should prompt a more comprehensive investigation and consideration of discussion with appropriate specialists.

Abbreviations: ALT, alanine aminotransferase; GGT, Gamma‐glutamyl transferase; MASH, Metabolic dysfunction‐associated steatohepatitis; MASLD, Metabolic‐dysfunction associated steatotic liver disease.

This strategy of testing for causes of hepatic steatosis diagnoses will lead to less testing than was recommended in the previous ESPGHAN Position Paper,^10^ though we recognise it will still lead to false positives and anxiety during investigations. The tests listed in Table 2 should only be performed in patients who have positive screening (i.e., raised transaminases) or are identified through increased echogenicity on US.

Identification of clinically significant portal hypertension is uncommon in paediatric MASLD at less than 2% in 10 years follow‐up, in one cohort^84^; therefore splenomegaly should be appropriately investigated as a red flag. Though it should be noted that data from adults shows that splenomegaly (without portal hypertension) is common and correlates with BMI,^81^ and splenic size demonstrated no correlation with degree of hepatic steatosis in one cohort of children.^85^

It is important to exclude Wilson′s disease in all patients investigated for MASLD. Serum caeruloplasmin <0.2 g/L has 95% sensitivity and 85% specificity for identification of Wilson′s disease.^82^ Yet, it should be noted that patients with WD can have normal caeruloplasmin levels.^86^ Therefore, clinicians should have a high index of suspicion for Wilson′s disease and perform comprehensive assessment (including at least urinary copper excretion and ophthalmological assessment with or without liver biopsy) in children with any red flags or a caeruloplasmin <0.2 g/L.^82^

Patients who do not meet these criteria (e.g., ≤8 years of age, not overweight [BMI *z*‐score <+1]) or have other ‘red flags′ (Table 3) should have a more detailed investigation, including screening for inborn errors of metabolism (Figure 2 and Table S3). Elevated liver enzymes are common in paediatric MASLD,^68^ however marked elevation (e.g., ALT ≥ 150 IU/L) may be an indicator of an alternative cause of hepatitis. Similarly, GGT ≥ 60 IU/L is more likely to be associated with advanced MASLD (stage 3–4 fibrosis),^17^, ^68^, ^87^ or an alternative diagnosis. The precise extent of investigation will depend on the clinical context and is at the discretion of a paediatrician with expertise in the management of paediatric liver disease. Liver biopsy may be used as a diagnostic test, but its place in the diagnostic pathway will vary (e.g., before or after genetic testing).

To diagnose MASLD, there should be evidence of hepatic steatosis (the accuracy of each modality for detection of steatosis is discussed in depth below). Next, test for other causes of steatosis (Table 2), and then children must meet cardiometabolic criteria (Table 4) that reflect metabolic dysfunction. This primarily requires a child to have an elevated BMI (*z*‐score ≥+1) or waist circumference. Different BMI cut‐offs are recommended for screening MASLD (in children with obesity, i.e., *z*‐score ≥+2) than for diagnosis (i.e., *z*‐score ≥+1) to mitigate unnecessary investigation of well children who are overweight (at the screening stage), while facilitating diagnosis of MASLD by non‐specialists (at the diagnosis stage). MASLD can be diagnosed in children with normal liver enzyme levels.

**Table 4:** Cardiometabolic criteria for the diagnosis of paediatric MASLD.

Criterion	Detail
Elevated BMI or waist circumference (*required*)	BMI ≥85th centile for age‐sex (*z*‐score ≥+1), or waist circumference ≥95th (ethnicity‐adjusted) centile
With or without	
Raised glucose	Fasting serum glucose ≥5.6 mmol/L (≥ 100 mg/dL), or
	Random glucose ≥11.1 mmol/L (≥ 200 mg/dL), or
	2‐h post‐load glucose ≥7.8 mmol/L (≥ 140 mg/dL), or
	Diagnosis of type 2 diabetes
Hypertension	<13 years: BP ≥95th centile, or ≥130/80 mmHg (whichever is lower); ≥13 years: BP ≥ 130/85 mmHg; or Antihypertensive treatment
Raised plasma triglycerides	<10 years: plasma triglycerides ≥1.15 mmol/L (≥100 mg/dL); ≥10 years: plasma triglycerides ≥1.7 mmol/L (≥150 mg/dL); or Lipid lowering treatment
Low plasma HDL	Plasma HDL ≤ 1.0 mmol/L (≤40 mg/dL), or lipid lowering treatment

*Note*: Children require the presence of an elevated BMI (at least overweight i.e., *z*‐score ≥ +1), or waist circumference. Additional criteria may support the diagnosis. Abbreviations: BMI, body mass index; BP, blood pressure; HDL, high‐density lipoprotein cholesterol; MASLD, metabolic dysfunction‐associated steatotic liver disease.

While MASLD in non‐overweight (BMI *z*‐score <+1) individuals is well described, it is less common, and the diagnosis should be made by a clinician familiar with the condition.^88^ Other supporting cardiometabolic criteria of the diagnosis (insulin resistance, hypertension and dyslipidaemia^1^, ^24^) should be present for a specialist to make a diagnosis of MASLD in a non‐overweight (BMI *z*‐score <+1) child.

The nomenclature change from NAFLD to MASLD has led to the recognition of combined metabolic and alcohol‐related liver disease (MetALD) as a distinct entity.^24^ The quantity and patterns of alcohol consumption in young people with MASLD are not well characterised; therefore, it remains important to routinely enquire about alcohol use in all adolescents diagnosed with MASLD.^58^

#### 4.1.1 Recommendations

4. Diagnosis of MASLD requires demonstration of steatosis on imaging or histology (in an appropriate clinical context). (LoE 3; 97%; strong consensus)
5. All children with suspected MASLD should have, as a minimum, first‐line investigations performed (Table 2). (LoE 3; 100%; strong consensus)
6. Further diagnostic investigations should be performed in patients who have any red flags (Table 3). (LoE 4; 100%; strong consensus)
7. Diagnosis of MASLD in non‐overweight (BMI *z*‐score <+1) children should be established by a specialist in the presence of both steatosis and metabolic dysfunction following thorough investigation. (LoE 3; 100%; strong consensus)

### 4.2 Assessment of comorbidities

The new definition of MASLD emphasises the bidirectional relationships of steatosis, cardiovascular diseases, diabetes and obesity.^24^ While the primary role of the gastro‐hepatologist is assessment of liver disease, patients may have undiagnosed comorbidities that need specific management (Table 5). If any of these screening tests are suggestive of comorbidities then follow‐up investigations (e.g., Sleep‐Related Breathing Disorder scale of the Paediatric Sleep Questionnaire [SRBD‐PSQ],^89^ oral glucose tolerance test^90^) can be performed, combined with seeking further specialist advice as necessary. Patients who have evidence of depression, anxiety, or other mental health disorders should be referred to appropriate specialists for further assessment and management.

**Table 5:** Comorbidities to screen for in patients with paediatric MASLD.

Comorbidity	Screening tests
Type 2 diabetes^6^	HbA1c, or fasting glucose^88^
Systemic (arterial) hypertension^12^	Blood pressure^89^
Dyslipidaemia^55^	Plasma lipid profile^90^
Obstructive sleep apnoea^91^	Sleep history^87^
Renal dysfunction^92^	Serum creatinine and urinary albumin‐creatinine ratio^93^
Polycystic ovarian syndrome^94^, ^95^	History for oligo‐amenorrhoea, examination for hyperandrogenism, ultrasound
Depression and anxiety^96^	Patient Health Questionnaire (PHQ9) and Generalised Anxiety Questionnaires (GAD7)^97^

*Note*: Children with MASLD are at increased risk of these conditions and should be screened for them every 1–2 years.

Abbreviation: MASLD, metabolic dysfunction‐associated steatotic liver disease.

Abnormal glucose tolerance (especially prediabetes) is highly prevalent among children/adolescents with biopsy‐proven MASLD. A cross‐sectional study showed that 11.1% of adolescents and 15.8% of young adults had impaired fasting glucose or prediabetes.^91^ Adolescent‐onset MASLD confers a 2.7 hazard ratio for onset of type 2 diabetes over a median of 8 years,^9^ which has been replicated in multiple other cohorts.^51^, ^92^, ^93^

#### 4.2.1 Recommendations

8. Paediatricians should assess for associated comorbidities (e.g., insulin resistance, type 2 diabetes, dyslipidaemia, arterial hypertension, kidney disease, sleep apnoea, polycystic ovary syndrome, depression and anxiety) in children with MASLD, at initial diagnosis and during the follow‐up. (LoE 3; 100%; strong consensus)

### 4.3 When to refer to a liver specialist?

Referral to a paediatric gastroenterologist or hepatologist for patients with MASLD is important in certain clinical situations to ensure accurate diagnosis and management. With a prevalence of 7%–8%, it is not possible (and also not required) for all children with MASLD to be seen by specialist paediatric gastro‐hepatologists. Children who are ≥8 years old and overweight (*z*‐score ≥+1) and do not have any red flags can be diagnosed with MASLD by non‐specialists (following appropriate investigation and exclusion or alternative or concurrent diagnoses).

The main roles of specialists are: ^1^ establishing diagnosis in atypical cases or diagnostic uncertainty; ^2^ identifying children at higher risk of advanced liver disease; and ^3^ initiating pharmacotherapy (or clinical trials, where available).

Indications for referral for diagnostic uncertainty are described above (Figure 2). The presence of risk factors for disease progression supports referral for further investigations and specialist management. The best markers for indicating children at higher risk of advanced liver disease are: liver enzymes (discussed in further detail below),^17^, ^38^, ^68^ severity of insulin resistance,^14^, ^38^, ^51^, ^93^ and family history of advanced fibrosis^94^, ^95^ Therefore, patients without red flags should be referred to specialist services if, after at least 6 months follow‐up, they have a persisting diagnosis of MASLD and any of the following:

o ALT ≥ 80 IU/L.^72^
o Pre‐diabetes or type 2 diabetes.^51^
o First‐degree relative with advanced liver fibrosis.^94^, ^95^

#### 4.3.1 Recommendations

9. Refer patients with red flags (Figure 2 and Table 3) to a specialist. (LoE 3; 100%; strong consensus)
10. In the absence of red flags, refer children with MASLD at increased risk of advanced liver disease (ALT ≥ 80 IU/L, persistently abnormal ALT despite weight loss, pre‐diabetes or type 2 diabetes, first‐degree relative with advanced liver fibrosis) to a specialist. (LoE 3; 100%; strong consensus)

## 5 NON‐INVASIVE INVESTIGATIONS

Following referral to a specialist service, further investigations are performed to determine MASLD severity. Here, we assessed the ability of each modality to diagnose and stage MASLD in children against liver biopsy as the reference standard. The validity of liver biopsy is considered below.

### 5.1 Liver enzymes for identification of MASLD

Elevated serum ALT, aspartate transaminase (AST) and GGT are frequently present in cohorts of paediatric patients with histologically confirmed MASLD.^15^, ^38^, ^48^, ^96^, ^97^, ^98^ However, they are not specific to MASLD^71^ and there is wide variability. The diagnostic accuracy of liver enzymes is described in detail under ‘Screening′ above.

GGT may be elevated in MASLD even in the absence of a cholangiopathy or alcohol consumption.^99^ GGT correlates strongly with insulin resistance^100^ and is also associated with markers of oxidative stress,^101^ which are reciprocally increased in steatohepatitis and insulin resistance,^102^ although the precise molecular pathways remain incompletely defined.

### 5.2 Liver enzymes for staging of MASLD

Across all identified cohorts with liver histology, higher liver enzymes were associated with a greater risk of steatohepatitis and higher stages of fibrosis.^14^, ^15^, ^48^, ^67^, ^68^, ^72^, ^98^ Children with ALT > 80 IU/L had twice the incidence of steatohepatitis compared to those with ALT < 80 IU/L in one study (41% vs. 21%).^72^ ALT > 100 IU/L has been associated with F3‐4 in multiple cohorts.^14^, ^16^, ^68^ This is in contrast to adults, where there is a limited relationship between liver enzymes and the histological stage.^103^ Nevertheless, ALT levels alone are not accurate for ruling in or ruling out fibrosis. In one study, the prevalence of advanced fibrosis in children with normal ALT, mildly elevated ALT (mean 37 IU/L) and highly elevated ALT (mean 123 IU/L) was 12% (2/17), 9% (7/74) and 15% (57/392), respectively.^67^ In another study, an ALT cut‐off of 50 IU/L had an 87% sensitivity but only 44% specificity for diagnosing advanced fibrosis,^68^ which may be improved by using composite models (discussed below).^14^, ^15^

### 5.3 Liver enzymes for monitoring of MASLD

Across all paired‐biopsy studies of MASLD in children, a reduction in liver enzymes has been associated with histological improvement.^17^, ^21^, ^38^, ^96^ A reduction of at least 40% in ALT and 20% in GGT levels was associated with composite histological improvement in patients receiving placebo,^17^, ^87^ but not necessarily fibrosis regression or resolution of steatohepatitis. Similarly, an increase of ≥20% in both ALT and GGT was associated with histological progression, however the accuracy of these cut‐offs has not yet been established in European populations. The meaning of smaller changes has not been well documented, and intra‐person variability should warrant caution in interpreting changes between 2 time points. In addition, weight loss and improvement in other markers of metabolic health (e.g., triglycerides) are consistently associated with reductions in liver enzymes.^22^, ^104^, ^105^ Conversely, rising GGT and the development of type 2 diabetes are associated with histological progression (steatosis, ballooning, lobular inflammation and fibrosis) of MASLD.^38^, ^87^ Overall, persistent and clinically meaningful (≥20%) changes in ALT and GGT are useful indicators of disease activity in paediatric MASLD, particularly when the magnitude of change is greater than 40% and occurs alongside change in other markers of metabolic health (e.g., HbA1c and triglycerides).

#### 5.3.1 Recommendations

11. ALT and GGT are suitable tools for identification and monitoring of progression/regression of MASLD in children. (LoE 3; 91%; consensus)

## 6 US

### 6.1 Conventional B‐mode for identification and grading of steatosis

US is relatively inexpensive, widely available, safe and quick. Increased brightness of the liver parenchyma (‘echogenicity′) compared to normal renal parenchyma is indicative of steatosis of the liver, though its appreciation is highly operator‐dependent.^106^ The utility of US B‐mode to identify steatosis regression or progression has not been studied. US is inaccurate for the assessment of steatohepatitis and fibrosis in MASLD.^107^ Accordingly, US is not suitable as a stand‐alone screening (as discussed above) or staging tool for paediatric MASLD.

Scoring systems have been developed that systematically score the US appearance of steatosis, improve accuracy and provide a semiquantitative assessment of steatosis severity.^108^ Pooling data from five studies^62^, ^109^, ^110^, ^111^ (*n* = 309) comparing US to MRI or histology for diagnosis of any steatosis showed sensitivity of 88% and specificity of 51% when using US score ≥1 and a sensitivity of 60% and specificity of 90% using US score ≥2 (Table S4). Therefore, a US score ≥2 makes MASLD highly likely; a US score of 0 generally excludes MASLD but hepatic steatosis can occur in the absence of US findings. When using US as a diagnostic tool, a US score ≥2 is most appropriate to limit the false‐positive rate, albeit at the cost of identifying only 60% of those affected.^107^, ^111^, ^112^

### 6.2 US quantification techniques for identification and grading of steatosis

To address the limitations of conventional B‐mode US, several quantitative US‐based techniques for steatosis assessment have been proposed including US beam attenuation,^113^ hepatorenal index,^114^ and shear‐wave dispersion^19^ (Table S5).

Although studies reported superior accuracy than conventional B‐mode, no prospective head‐to‐head comparison has been performed. There were substantial limitations in methodology and techniques reported. The largest study (*n* = 140) reported 84% sensitivity and 93% specificity to differentiate no from any steatosis using US attenuation.^113^

#### 6.2.1 Recommendations

12. US B‐mode of at least moderate severity (score ≥2 out of 3) can be used to diagnose hepatic steatosis in children. (LoE 3; 97%; strong consensus)
13. US is not a suitable method to assess steatohepatitis or the degree of fibrosis in children. (LoE 3; 100%; strong consensus)

### 6.3 Controlled attenuation parameter (CAP™)

CAP™ is a specific form of US attenuation that is used to indicate the degree of steatosis. It has been developed on the FibroScan© device, although other US‐based techniques and devices are developing comparable tools. Several studies of CAP™ in children report cut‐off values ranging from 225 to 281 dB/m to diagnose hepatic steatosis in comparison to histology or MRI^112^, ^115^, ^116^, ^117^, ^118^, ^119^, ^120^, ^121^, ^122^, ^123^, ^124^ (Table S6). Values > 280 dB/m were consistently associated with steatosis. In a study of children with biopsy‐confirmed MASLD, CAP™ > 259 dB/m strongly correlated with steatosis grade (with >259 dB/m having an AUROC of 0.98 for the presence of any steatosis).^120^ However, it is still unclear what the optimal diagnostic cut‐off should be.^115^, ^116^

The correlation between CAP™ and hepatic steatosis measured by MRI is decreased in individuals with higher body mass index and increased abdominal wall thickness,^117^, ^121^, ^122^, ^123^ suggesting lower accuracy in severe obesity (BMI *z*‐score ≥+3).

There are no studies in children evaluating the utility of change in CAP™ to measure changes in hepatic steatosis over time, though CAP™ does decrease in response to weight loss.^105^ There is no evidence to support the magnitude of change in CAP™ that would reflect a histological improvement in MASLD.

#### 6.3.1 Recommendations

14. In patients with a diagnosis of MASLD, CAP may be a suitable adjunct for the quantification of steatosis. (LoE 4; 100%; strong consensus)
15. CAP > 280 dB/m is strongly associated with hepatic steatosis, but further evidence is needed to identify optimal cut‐off values for diagnosis and grading of steatosis. (LoE 4; 97%; strong consensus)

## 7 LIVER STIFFNESS MEASUREMENT (LSM)

### 7.1 Transient elastography

Vibration‐controlled transient elastography (VCTE [TE]) is a non‐invasive US‐based technology used to derive a liver stiffness measurement as a surrogate of hepatic fibrosis. It is influenced by multiple factors, including steatohepatitis. High‐quality data in adults indicate that (1) LSM > 10 kPa is suggestive of advanced fibrosis and LSM > 15 kPa is highly suggestive of advanced chronic liver disease, (2) LSM correlates with liver‐related clinical events^125^ and has a prognostic value that is equal to fibrosis stage on biopsy and (3) changes in LSM of >30% over time (progression and regression) impact prognosis in either direction.^126^

Six studies have compared histological fibrosis to TE in children with MASLD (Table S7): one cohort of 84 children found no correlation between TE and fibrosis stage,^19^ a further study found AUROC 0.7 to distinguish F0‐2 versus F3‐4 (Ishak stage),^120^ and one cohort (in two papers) reported 100% sensitivity and specificity.^127^ Three further cohorts of mixed aetiology (i.e., not exclusively MASLD) reported 71%–83% sensitivity to detect advanced fibrosis,^128^, ^129^ with one reporting AUROC 0.83 to distinguish no‐moderate fibrosis versus severe with a cut‐off of 10.6 kPa.^130^ Across all studies, TE has a limited ability to distinguish between the early and intermediate stages of fibrosis. TE > 10 kPa was more consistently associated with more advanced stages of fibrosis, and <5.5 kPa represented values within the reference range, hence ‘normal’.^129^, ^131^ A meta‐analysis of data from over 1700 children found an ULN to be 5.5 kPa.^132^

The technique is quick, easy and has acceptable reproducibility; thus it can be performed as point‐of‐care.^133^ Factors influencing liver stiffness measurements include a non‐fasted state, degree of steatosis, hepatic inflammation, cholestasis, or congestion.^129^ Failure can be due to ascites or obesity. To reduce the failure rate in patients with severe obesity an XL probe should be used. In contrast to adult data,^134^ cut‐off points for different stages of fibrosis or cirrhosis have not been precisely validated (as summarised in Table S7) and further studies are needed to establish TE accuracy in paediatric MASLD.

#### 7.1.1 Recommendations

16. Transient elastography may be useful to exclude significant liver fibrosis (<5.5 kPa) or suggest advanced chronic liver disease (>10 kPa) in children with MASLD. (LoE 3; 94%; strong consensus)
17. In children with MASLD, there is insufficient evidence to accurately stage fibrosis with transient elastography liver stiffness measurements between 5.5 and 10 kPa. (LoE 3; 100%; strong consensus)

### 7.2 Shear‐wave elastography (SWE)

SWE can be integrated into a conventional US machine and is able to measure the stiffness of hepatic tissue using real‐time localisation in the liver. Compared to VCTE, it does not necessitate an additional device (although point‐of‐care applications are being developed), allows for real‐time visualisation of the area measured, and does not use a mechanical impact to create a pulse wave. SWE has variable accuracy in detecting hepatic fibrosis, with cut‐off values to detect hepatic fibrosis ranging from 1.24 to 1.89 m/s.^135^, ^136^, ^137^, ^138^, ^139^, ^140^, ^141^ Published studies, however, include small numbers of patients, with a minority having MASLD, and use a variety of methodologies (Table S8).

Similar to transient elastography, SWE values are reported to be increased in the presence of inflammation in most,^138^, ^139^, ^142^ but not all,^141^ studies. The use of SWE is limited by higher technical failure rates in patients with obesity.^141^

#### 7.2.1 Recommendations

18. Currently, there is insufficient evidence to recommend shear‐wave elastography for use in children with MASLD. (LoE 4; 97%; strong consensus)

### 7.3 MRI‐PDFF

MRI‐PDFF has emerged as the gold standard in the non‐invasive assessment of liver steatosis,^12^ outperforming US‐based steatosis grading and CAPTM.^143^ There is compelling evidence from multiple, independent liver biopsy studies in children that MRI liver lipid content correlates with histological steatosis (*n* > 300).^144^, ^145^, ^146^, ^147^ MRI‐PDFF is commonly used as a non‐invasive reference standard in clinical studies^148^ and there is almost complete correlation between MRI‐PDFF and magnetic resonance spectroscopy (MRS) modalities.^149^ MRI‐PDFF is suitable for monitoring longitudinal changes in liver lipid content in children with MASLD.^145^ Importantly, the continuous PDFF (%) of the liver is not identical to the histological evaluation of liver steatosis (% of hepatocytes with lipid droplets) and different cut‐offs in the definition of steatosis grades may bias the comparability of study results. The principal limitations of MRI‐PDFF (and MR elastography) are its limited availability, high cost and not being tolerated by some patients, including the requirement for general anaesthetic.

MRI‐PDFF > 20% is associated with at least 50% risk of fibrosis and 30%–35% of steatohepatitis.^18^, ^20^ MRI‐PDFF also shows reproducible dynamic changes with histological disease improvement, where a ≥30% reduction (e.g., 15% liver fat to 10% liver fat) is more often associated with resolution of steatohepatitis in adults.^150^ While there is no similar paired biopsy‐MRI data in children, reduction in MRI‐PDFF liver fat correlates with decreases in ALT and GGT.^20^, ^151^

#### 7.3.1 Recommendations

19. MRI‐PDFF has a high diagnostic accuracy for the quantification of hepatic lipid content in paediatric patients with MASLD and can serve as a non‐invasive reference standard for the assessment and monitoring of steatosis, although it is typically not available in routine clinical practice. (LoE 2; 100%; strong consensus)

### 7.4 MRE

MRE‐measured liver stiffness is used to estimate fibrosis stage. Data from paediatric and adult cohorts indicate that MRE‐measured liver stiffness is not affected by hepatic steatosis grade in patients with MASLD.^152^, ^153^ MRE captures 2D stiffness maps of the whole liver, thereby potentially reducing the effect of sample error compared to US‐based elastography or liver biopsy^154^ in addition to better inter‐reader agreement.^155^ MRE‐measured liver stiffness has been widely validated as a marker of fibrosis in adults with MASLD, yielding the highest AUC values compared to other elastography methods.^156^, ^157^

Three prospective studies have investigated MRE in paediatric cohorts with biopsy‐proven MASLD (Table S9).^18^, ^142^, ^144^ MRE‐measured liver stiffness showed similar high diagnostic accuracy for the detection of advanced fibrosis in two cohorts (Area Under the Receiver Operating Characteristic curve [AUROC] > 0.87) while it was less useful in the detection of any fibrosis (AUROC 0.77–0.79).^18^, ^142^ The third cohort did not demonstrate any association between liver stiffness and histological fibrosis.^144^

No established reference values for MRE in children with liver disease are available at the moment, but they seem to correspond to adult reference values.^158^

Other MRI‐based markers have been developed to quantify liver fibrosis, including multiparametric MRI (mpMRI). The primary parameter used in mpMRI is iron‐corrected longitudinal relaxation time T1 (cT1). The diagnostic value of cT1 in the evaluation of MASLD has been studied only in adults to date.^159^ Yet, since steatosis, inflammation, and fibrosis all influence cT1, its role in risk stratification in paediatric MASLD needs to be further studied.

#### 7.4.1 Recommendations

20. MRE may be useful for identification of advanced fibrosis in paediatric MASLD, although validation of optimal cut‐offs is needed, and it is typically not available in routine clinical practice. (LoE 3; 100%; strong consensus)

### 7.5 Composite non‐invasive scores and serological biomarkers

Several non‐invasive serological biomarkers and composite scoring algorithms have been proposed for stratification of paediatric MASLD, mainly relating to liver fibrosis.

### 7.6 Prediction of fibrosis stage

Simple non‐invasive scores (e.g., FIB‐4 [Fibrosis‐4 index], NAFLD Fibrosis Score, APRI [aspartate aminotransferase to platelet ratio index]) provide reliable risk stratification in adults with MASLD.^160^ However, these do not perform well for children with MASLD,^14^, ^68^ as age is a major factor in their derivation, and the underlying prevalence of end‐stage liver disease is substantially higher in adult cohorts with MASLD. The paediatric NAFLD fibrosis index (PNFI) and the paediatric NAFLD fibrosis score (PNFS) were both derived specifically for children with MASLD; however, initial reports of utility have not been replicated.^14^, ^68^ A recent analysis of 457 European patients with biopsy‐proven MASLD found that no existing scores showed diagnostic benefit over using ALT ≥ 50 IU/L as a cut‐off.^68^

Two recently developed composite scoring systems have potential utility: pFIB‐c^15^ and Fibro‐PeN.^14^ pFIB‐c was created to exclude ≥F2 stage fibrosis, having been derived in a European cohort of patients with obesity. It has lower accuracy in histological cohorts (AUROC 0.71–0.77).^15^ Fibro‐PeN was created to identify F2‐4 stage fibrosis, derived from 1055 children in the USA with biopsy‐proven MASLD and had AUROC 0.79, though 70% of participants were of Hispanic ethnicity, which is not typical for European cohorts.^14^

#### 7.6.1 Recommendations

21. FIB‐4, PNFI, PNFS and APRI are not recommended for diagnosis or stratification in children with MASLD. (LoE 2; 100%; strong consensus)
22. pFIB and Fibro‐PeN require further validation prior to their use in clinical practice. (LoE 3; 100%; strong consensus)

### 7.7 Enhanced liver fibrosis (ELF) test

The ELF score combines 3 serum markers of extracellular matrix remodelling and fibrogenesis: hyaluronic acid (HA), the N‐terminal pro‐peptide of collagen type III (PIIINP) and tissue inhibitor of metalloproteinase‐1 (TIMP‐1).^161^ The ELF test has been studied extensively in adults and shown to be a good marker of fibrosis with AUROC of 0.81 for the detection of F2‐4.^162^ However, ELF changes with pubertal growth in children.^163^

One cohort of paediatric MASLD reported AUROC > 0.95 for identification of advanced fibrosis (≥F3)^164^ across two publications.^165^ However, in two subsequent cohorts, there was no significant association found between ELF components and fibrosis stage.^166^, ^167^ One other cohort reported AUROC 0.7‐0.79 for identification of F2‐3 fibrosis^168^ and noted that a decrease in ELF correlated with improvement in steatohepatitis but not fibrosis.

### 7.8 Other biomarkers

Many other biomarkers of paediatric MASLD have been proposed, including cytokines,^169^, ^170^ cytokeratin‐18 fragment^166^, ^171^ and adipokines,^172^ however, none are in routine clinical use.

#### 7.8.1 Recommendations

23. There is insufficient evidence to recommend the use of ELF (or its components) in paediatric MASLD. (LoE 3; 100%; strong consensus).

### 7.9 Genetic testing

MASLD is a polygenic disorder with multiple genetic variants identified through genome‐wide association studies (GWAS) in adults, which play critical roles in disease development and progression.^173^, ^174^, ^175^, ^176^ These genes almost all contribute to either lipid storage in hepatocytes or insulin resistance.^177^

p.Ile148Met in PNPLA3 (rs738409C>G) is by far the most substantial contributor to the genetic risk of MASLD.^178^, ^179^ This variant confers a significant risk of mortality in adults with MASLD through increased liver‐related events.^180^ It has been consistently associated with more severe histology in children with MASLD.^6^, ^16^, ^181^, ^182^ This variant may contribute to the increased severity of MASLD in children of Hispanic ancestry, as it is carried at a higher frequency (51%) compared to 22% carriage in those of European ancestry.^183^

Other variants including those in or near genes *TM6SF2*, *MBOAT7*, *GCKR*, *HSD17B13* and *MTARC1* have all been associated with MASLD histology in children.^16^, ^98^, ^184^ However, none have been demonstrated to confer the same degree of worsening histology as p.Ile148Met in PNPLA3.

Genetic testing may play a role in the investigation of alternative causes of MASLD, including inborn errors of metabolism, Wilson′s disease, severe early‐onset obesity and insulin resistance syndromes.^10^, ^44^, ^185^ It may be particularly useful in children under 5 years old with hepatic steatosis, as well as in patients with red flags, or who have persistently raised liver enzymes despite weight loss.

#### 7.9.1 Recommendations

24. Children under 5 years old with hepatic steatosis should be investigated for genetic diseases. (LoE 2; 100%; strong consensus)
25. Genetic testing (targeted panels or whole‐exome) may play a role in investigating alternative causes of steatotic liver disease, particularly in lean individuals. (LoE 4; 100%; strong consensus)
26. Testing for p.Ile148Met in PNPLA3 and other common variants is currently not recommended in clinical practice. (LoE 3; 91%; strong consensus)

## 8 LIVER BIOPSY

### 8.1 Histological definitions of paediatric MASLD

Steatosis in more than 5% of hepatocytes is the minimum histological feature for diagnosis of MASLD.^186^ Paediatric MASLD may demonstrate an ‘adult′ phenotype: a pericentral pattern of steatosis and fibrosis with ballooning^187^, ^188^; however, pre‐ and peri‐pubescent children more often have periportal (zone 1) predominant steatosis, with portal inflammation, and fibrosis.^189^ These children often do not present with ballooned hepatocytes. These phenotypes have been described as ‘type 1′ and ‘type 2′ paediatric NAFLD, respectively^48^, ^190^ however, it is now more common to refer to zonal patterns.^38^, ^191^ Children with periportal predominant disease have been referred to as having ‘borderline zone 1 steatohepatitis′, due to the absence of ballooning and Mallory‐Denk bodies.^67^, ^192^ However, this is a full manifestation of steatohepatitis in this age group (Table 6).^190^

**Table 6:** Histological diagnosis of paediatric MASLD.

Histological diagnosis	Description
Hepatic steatosis ± zone 1 or 3 predominant	Lipid accumulation in >5% of hepatocytes ± most prominent in periportal (zone 1) or perivenular (zone 3) regions
Zone 1 predominant steatohepatitis	Fibrosis predominantly in the periportal region (zone 1). Hepatocyte ballooning is rarely observed.
Zone 3 predominant steatohepatitis	Hepatocyte ballooning and Mallory‐Denk bodies with or without pericellular/‐sinusoidal fibrosis, predominantly in the perivenular region (zone 3).

*Note*: Patients may demonstrate a zone 1 or 3 dominant pattern of disease. Diagnosis of MASH in children does not require the presence of ballooning or Mallory‐Denk bodies for zone 1 predominant disease, or if there is established zone 3 fibrosis.

Abbreviations: MASH, Metabolic dysfunction‐associated steatohepatitis; MASLD, Metabolic‐dysfunction associated steatotic liver disease.

It is unclear to what extent paediatric MASLD moves between portal and centrilobular dominant inflammation. This ‘paediatric phenotype’ of MASH is associated with younger age, male sex and more severe metabolic dysfunction.^48^ Moreover, periportal steatosis^193^ and portal inflammation^48^ are independently associated with advanced fibrosis. Genetic factors may also influence the development of zonal disease as PNPLA3 p.Ile148Met^181^, ^182^ and *HSD17B13* rs72613567T>TA variants are associated with differing severity of portal disease.^16^

Histological features of steatosis and ballooning are not unique to MASLD (they can be seen in Wilson′s disease, for example). Ballooning and Mallory Denk bodies are seen in a periportal location in chronic biliary disorders. Periportal steatosis can be a clue to alpha‐1 antitrypsin deficiency in the infant. As is always the case in liver biopsy interpretation,^194^ clinical correlation is essential for diagnosis.^1^, ^71^

The NASH CRN Staging is typically used for MASLD^195^; with additional assessment of portal inflammation.^190^, ^191^ Fibrosis is usually staged from 0 to 4 according to the NASH CRN system. Given the periportal disease in children, the NAFLD Activity Score (including intralobular but not portal inflammation) may not fully capture the severity of histology^48^, ^190^ and is not intended for diagnosis of steatohepatitis.^196^ These scoring systems have been applied to multiple cohorts internationally.^166^, ^182^, ^195^

There is limited data in children associating clinical end‐points with histological staging, unlike in adults.^188^ A single, retrospective study of adolescents and young adults (wherein 44% were <18 years) found that ‘NASH with or without fibrosis′ was associated with increased mortality.^32^ Data from RCTs demonstrates that progression of liver histology correlates with the severity of insulin resistance.^38^

#### 8.1.1 Recommendations

27. Histopathologic evaluation of MASLD in children should include a comprehensive assessment of the biopsy to discriminate between simple steatosis or steatohepatitis, including the assessment of portal inflammation and portal‐based fibrosis, as well as a description of the zonal distribution of steatosis. (LoE 4; 100%; strong consensus)
28. Where an experienced histopathologist has identified significant portal inflammation and/or fibrosis, the term ‘zone 1 steatohepatitis’ should be used (and not ‘borderline steatohepatitis’). (LoE 4; 97%; strong consensus)

### 8.2 Reliability and reproducibility

Liver biopsy is frequently described as the ‘gold standard′ for diagnosis and staging of MASLD in children.^45^ There is general agreement that adequate biopsies should be >2 cm and of ‘adequate width′. There are no studies in children that describe the impact of sampling and intra‐/inter‐observer variability. For adult MASLD, it is well described that steatosis is reliably determined, but there is greater variability in the assessment of fibrosis. It is well recognised that small specimens underestimate fibrosis, inflammation and, in particular, ballooning, and therefore of steatohepatitis.^197^, ^198^, ^199^, ^200^ There is sampling variability in adults with MASLD; in a study of 51 adults, 35% patients with F3 fibrosis had F0‐1 in a paired biopsy.^201^

### 8.3 Risks of liver biopsy in patients with MASLD

The risk of complications with percutaneous liver biopsy is higher in children (5%) as compared to adults (1%),^202^, ^203^, ^204^ though other studies describe <2% major complications.^205^, ^206^ In patients with obesity, consideration of US‐guided liver biopsy or transjugular biopsy is recommended over percutaneous ‘blind’ liver biopsy,^207^, ^208^ though this is not based on data. A single series of blind percutaneous liver biopsies in 30 children with obesity reported no major complications and no difference with individuals without obesity.^209^ There were no reported major adverse events across >700 liver biopsies as part of randomised controlled trials in paediatric MASLD.^21^, ^96^

#### 8.3.1 Recommendation

29. When biopsy is performed in children with MASLD, US‐guided technique should be used where possible. (LoE 4; 100%; strong consensus)

### 8.4 Role of biopsy in assessing treatment response

Liver biopsy is typically used as the reference standard for the assessment of treatment response. In one cohort (across three papers), weight loss >10% (with or without weight‐loss surgery) was associated with improvements in hepatic steatosis, inflammation and ballooning^210^, ^211^ and greater weight loss was associated with improvement in fibrosis stage.^104^ This is generally consistent with adult data, where 84% of patients had MASH resolution at 1 year after bariatric surgery, with no significant recurrence between 1 and 5 years.^212^ Fibrosis began to decrease by 1 year after surgery and continued to decrease until 5 years.

### 8.5 Indications for liver biopsy

Potential roles for liver biopsy include: diagnosis, risk stratification and deciding on treatment. There is uniform consensus across guidelines that liver biopsy is an appropriate test when aetiological diagnosis is unclear, particularly if autoimmune hepatitis (or Wilson′s disease) is suspected,^1^, ^45^, ^74^ which cannot be reliably diagnosed by other tests. It should be noted that autoantibody positivity is present in up to 28% of children with MASLD and raised immunoglobulin G in 17%.^80^ Anti‐liver‐kidney‐microsomal antibody positivity is not associated with MASLD.^80^ Therefore, suspicion of autoimmune hepatitis should be made in the appropriate clinical context.^83^

Risk stratification of children with MASLD is critical to manage demand, given an overall prevalence of 7%–8% and 52% in children with obesity.^43^ Moreover, fibrosis stage (particularly F2‐4) conveys important prognostic information about the risk of mortality.^32^, ^213^

However, practice in Europe has evolved since the last ESPGHAN Position Paper on MASLD,^10^, ^207^ which recommended liver biopsy for 6–12 months of persistently elevated liver enzymes despite lifestyle interventions. A survey of European paediatric gastro‐hepatologists found that more than 75% of respondents performed biopsy in fewer than 10% of patients.^13^ It is well established that liver enzymes remain persistently elevated in >50% of children with MASLD.^36^, ^38^ Therefore, raised transaminases alone are not a practical indication for biopsy.

At this time, there are no licensed therapies for paediatric MASLD, even though resmetirom is approved in the US and Europe for adults with F2‐3 and MASH.^214^ In addition, GLP‐1‐based therapies are already available for children and adults with obesity and/or insulin resistance.^215^, ^216^ Identification of patients with MASH and/or F2‐3 fibrosis will influence decisions to initiate treatment with such agents.

#### 8.5.1 Recommendations

30. Liver biopsy is an accurate tool for diagnosis and follow‐up/monitoring of paediatric MASLD (LoE 2; 90%; consensus)
31. Liver biopsy (including repeat biopsy) should be performed in cases of diagnostic uncertainty and/or findings of more advanced liver disease (based on clinical, biochemical, imaging, or transient elastography investigations). (LoE 4; 94%; consensus)
32. Liver biopsy may be used for risk stratification in high‐risk individuals to identify adolescents with fibrosing steatohepatitis to: (i) facilitate accurate follow‐up under adult services, (ii) initiation of drug treatment, or (iii) for clinical trials. (LoE 4; 100%; strong consensus)

### 8.6 Follow‐up

**Figure 3:**
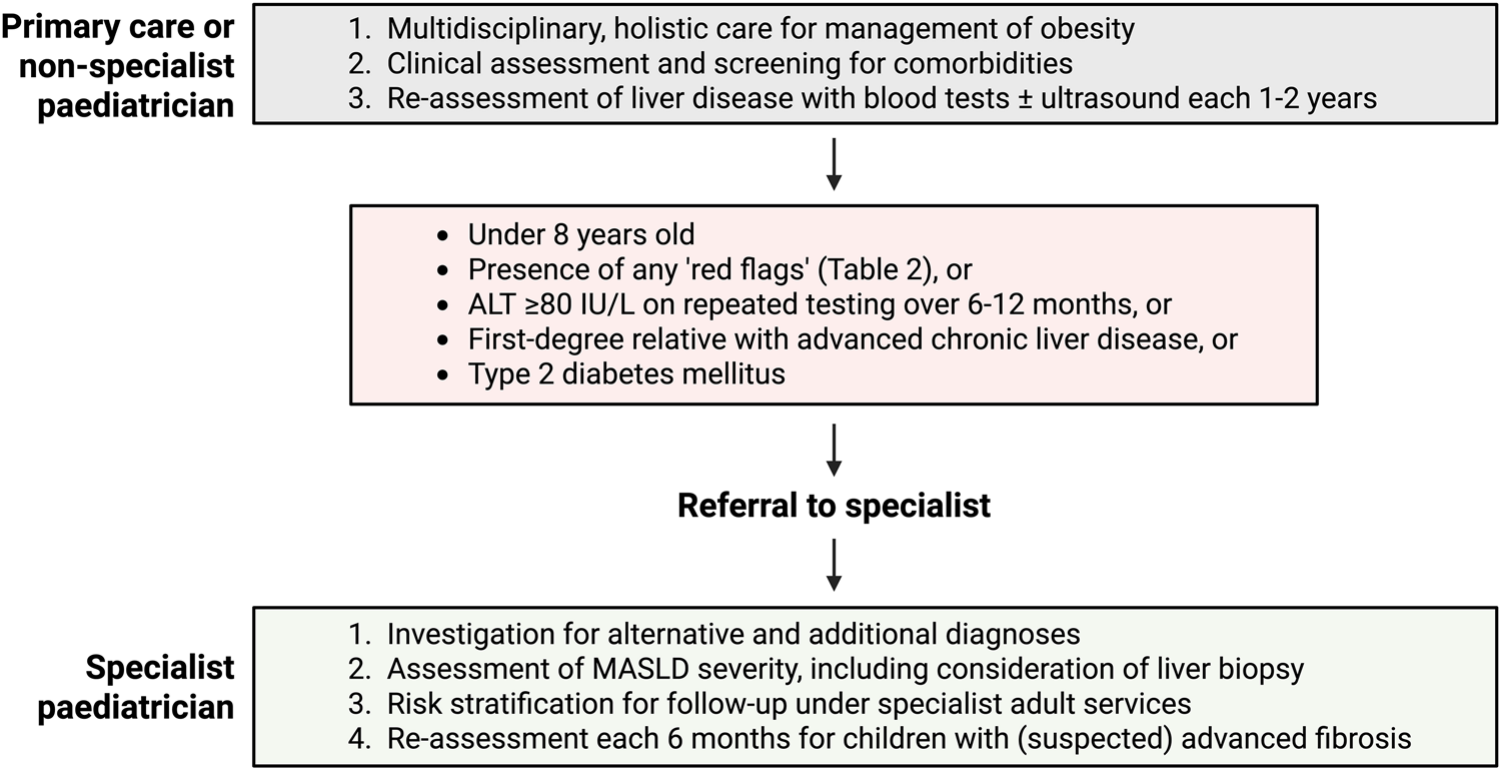
Monitoring and follow‐up of children with MASLD. Most children with MASLD will be managed by non‐specialists, focusing on holistic care to manage obesity. Patients should be referred to specialists in the presence of any red flags or signs suggesting more advanced disease. Paediatric gastro‐hepatology services will assess disease severity for risk stratification, targeted follow‐up and enrolment into clinical trials. MASLD, metabolic dysfunction‐associated steatotic liver disease.

As progression is variable but seems to occur in up to one‐third of patients, interval follow‐up to monitor progression of disease is indicated (Figure 3). Most patients should be cared for by non‐specialists, primarily focusing on the management of obesity. The presence of any red flags, age under 8 years, or other risk factors of more advanced disease (persistent ALT ≥ 80 IU/L, family history of advanced fibrosis, or type 2 diabetes) should prompt referral to a specialist.

Specialist services (typically paediatric gastro‐hepatologists) may utilise the non‐invasive tools described above (e.g., transient elastography ≥10 kPa) to help identify patients at risk of advanced disease.

Management of excess weight should be the focus of treatment. Weight loss of ≥10% body weight is associated with improvements in liver histology,^210^, ^211^ transient elastography and transaminases in children.^105^ Even more modest reductions in BMI (−0.1 kg/m^2^) can be associated with improvement in transaminases.^217^

Transitioning of young people with MASLD to adult services should be an individualised approach. Due to service constraints, most patients are likely to be followed‐up as adults by primary care or non‐hepatology specialists. It should be recognised that the focus of adult MASLD management in many European countries is identification of individuals with advanced fibrosis. However, routinely employed risk scores such as the FIB‐4 ^12^ are not recommended in patients <35 years of age as the inclusion of age in the formula might lead to false‐negative results.^68^ In preparation for transition, young people should have completed their diagnostic evaluation (including consideration of the need for liver biopsy), undergone VCTE assessment, and had a recent re‐evaluation of both liver disease status and extrahepatic comorbidities. Paediatric and adult centres should engage to establish criteria (e.g., persistent ALT ≥ 80 IU/L, or VCTE ≥ 8 kPa, or biopsy‐confirmed steatohepatitis or ≥F2 fibrosis) for follow‐up of transitioned patients under specialist adult services.^218^ The precise age and ALT or VCTE cut‐off values for transition to specialist adult services will vary with local and national health service availability.

#### 8.6.1 Recommendations

33. Each child or adolescent with MASLD needs holistic care within a multidisciplinary team for the management of metabolic dysfunction (LoE 3; 100%; strong consensus)

## 9 CONCLUSION

Paediatric MASLD is a highly prevalent, chronic, progressive disorder where patients are at particular risk of developing type 2 diabetes. Unlike in adults with MASLD, ALT is a suitable test for screening and risk stratification in paediatric MASLD, but demonstration of steatosis (typically with US) is required for diagnosis. Children over 10 years old with obesity (BMI *z*‐score ≥+2) should be screened for MASLD using an ALT of ≥30 IU/L. Children with features of metabolic dysfunction can be screened for MASLD at any age. Most patients can be diagnosed and managed without requiring input from specialist paediatric gastro‐hepatologists. However, all patients need investigation for alternative causes of raised liver enzymes and hepatic steatosis. In addition, patients with red flags should undergo extended investigation and all patients under 8 years of age should be referred to a specialist. Further evidence is needed to understand how to routinely implement non‐invasive diagnostic tools for children with MASLD.

## CONFLICT OF INTEREST STATEMENT

Ruth De Bruyne is funded as senior clinical investigator by FWO Flanders (1843824 N) and has received consulting fees from Mirum Pharma and Ipsen. Jake P. Mann is funded by NIHR ACL (CL‐2022‐09‐005), Royal Society (RG\R1\241312), BSPGHAN‐Guts (BSPGHAN2023_01), ESPGHAN, Royal College Physicians Dame Sheila Sherlock Bursary, and Little Princess Trust (CCLGA 2024 10 Mann) and has received consulting fees from Novo Nordisk, Eli Lilly and Invetiva. The remaining authors declare no conflicts of interest.

## Supporting information

Supplementary File 1. Summary of all recommendations from this position paper.

Figurementary Figure 1: Radarplot of the final consensus reached per statement/recommendation. 31 members of the Special Interest Group on MASLD (SIG‐MASLD) were invited to vote on agreement with statements and recommendations via Qualtrics using a four‐point scale (“totally agree”; “somewhat agree”, “somewhat disagree”, “disagree”). Consensus was defined as at least 80% of the members responding with either “agree” or “somewhat agree”, whilst ≥95% agreement was classified as ‘strong consensus′. Supplementary figure 1 visualizes the final degree of consensus reached per statement/recommendation. “Totally agree” equals the number of participants who have voted “totally agree”; “Agree” equals the number of participants who have voted “totally agree + somewhat agree”.

Supplementary Table 1: results of the voting on the statements and recommendations. 31 members of the Special Interest Group on MASLD (SIG‐MASLD) contributed to 2 consecutive voting rounds. All participating members were invited to vote on agreement with statements and recommendations via Qualtrics using a four‐point scale (“totally agree”; “somewhat agree”, “somewhat disagree”, “disagree”). Consensus was obtained for all statements and recommendations after the first voting round. Based on the level of agreement and the feedback provided during the first voting round, 15 statements and recommendations were reformulated and underwent a second voting round. The results are shown in Table 1a (first voting round) and Table 1b (second voting round, statements and recommendations in bold were adapted based on the feedback from the first voting round and revoted upon).

Supplementary Table 2: Aspects of medical history and physical examination which can help in risk assessment for paediatric MASLD.

Supplementary Table 3. Extended investigations for patients with hepatic steatosis. The precise extent, timing, and order of investigations will be based on the clinical context and should be determined in conjunction with relevant specialists (e.g. inherited metabolic disease specialists). The Genomics England testing panels (https://nhsgms-panelapp.genomicsengland.co.uk) are given as examples of gene panels used in the assessment of patients, given the relevant phenotype.

Supplementary Table 4. Studies investigating ultrasound for liver steatosis.

Supplementary Table 5. Studies investigating quantitative ultrasound for liver steatosis.

Supplementary Table 6. Studies comparing CAP for liver steatosis to MRI‐PDFF or liver biopsy.

Supplementary Table 7. Studies comparing VCTE for liver fibrosis to MR elastography or liver biopsy.

Supplementary Table 8. Studies comparing SWE for liver fibrosis to MR elastography or liver biopsy.

Supplementary Table 9. Studies comparing MR elastography to liver biopsy.
