# Supplementary material for: European Society for Pediatric Gastroenterology, Hepatology and Nutrition (ESPGHAN) steatotic liver disease special interest group position paper on screening, diagnosis and investigation of paediatric metabolic dysfunction‐associated steatotic liver disease
Source: J Pediatr Gastroenterol Nutr. 2026 Jun 30;83(3):555–76. doi: 10.1002/jpn3.70490 (PMC13539658; doi:10.1002/jpn3.70490)
Supplement: Supplementary file 1 [file JPN3-83-555-s001.docx]

Supplementary Table 7. Studies comparing VCTE for liver fibrosis to MR elastography or liver biopsy

| **Author,**  **Year** | **Location** | **Sample size (M/F)** | **Age: mean (SD) or median (IQR)** | **Exclusion criteria** | **Comparator** | **Fibrosis stages** | **AUC and cut-off** |
| --- | --- | --- | --- | --- | --- | --- | --- |
| Alkhouri, 2013 | Rome, Italy | 67  (46/21) | 8.4-9.3 | Unsuccessful VCTE | Liver biopsy | 15% F0-F1  85% F2-F3 | AUC ≥F2: 1.0, cut-off >8.6 kPa |
| Chaidez, 2022 | Colorado, US | 116 NAFLD  (84/32) | 13.7 (3.7) | Liver transplantation, ascites, abdominal skin disease | Liver biopsy | Ishak 0-2: 98  Ishak 3-4: 18  Ishak 5-6: 0 | AUC Ishak ≥3: 0.70 |
| Fitzpatrick, 2013 | London, UK | 101 (37 NAFLD) | 13.5 (11.9–14.6) | Too small for M-probe, ascites, extrahepatic fibrotic disease | Liver biopsy | 6% F0  29% F1  25% F2  33% F3  7% F4 | AUC ≥F2: 0.79, cut-off >6.1 kPa  AUC ≥F3: 0.87, cut-off >6.9 kPa |
| Lee, 2018 | Boston, US | 267 (31 SLD)  (143/124) | Median 13 | Ascites, BMI >40, pregnancy, implantable cardiac device, Fontan | Liver biopsy | 17.6% F0  32.2% F1  19.5% F2  16.9% F3  13.9% F4 | Derivation cohort:  AUC ≥F3: 0.85, cut-off >8.6 kPa  AUC ≥F4: 0.87, cut-off >11.5 kPa |
| Ravanbakhsh, 2024 | Los Angeles, US | 77 MASLD  (57/20) | 14 (12-16) | Concurrent liver disease  VCTE, MRE or biopsy unavailable | Liver biopsy | 10% F0  34% F1  35% F2  19% F3  1% F4 | AUC ≥F3: 0.75 |
| Teufel-Schäfer, 2021 | Heidelberg, Germany | 75 (10 NAFLD)  (42/33) | 12.3 | Hemochromatosis, bile duct atresia, acute liver failure | Liver biopsy | 33% F0-F1  47% F2  20% F3-F4 | AUC ≥F3: 0.83, cut-off >10.6 kPa |
