# Supplementary material for: European Society for Pediatric Gastroenterology, Hepatology and Nutrition (ESPGHAN) steatotic liver disease special interest group position paper on screening, diagnosis and investigation of paediatric metabolic dysfunction‐associated steatotic liver disease
Source: J Pediatr Gastroenterol Nutr. 2026 Jun 30;83(3):555–76. doi: 10.1002/jpn3.70490 (PMC13539658; doi:10.1002/jpn3.70490)
Supplement: Supplementary file 2 [file JPN3-83-555-s002.docx]

Supplementary Table 6. Studies comparing CAP for liver steatosis to MRI-PDFF or liver biopsy

| **Author,**  **Year** | **Location** | **Sample size (M/F)** | **Age: mean (SD) or median (IQR)** | **Exclusion criteria** | **Comparator** | **Steatosis grades** | **AUC and cut-off** |
| --- | --- | --- | --- | --- | --- | --- | --- |
| Anand, 2022 | New Delhi, India | 108  (72/36) | 12.4 (1.9) | Concurrent liver disease, genetic or endocrine obesity, systemic illness, treatment with metformin or vitamin E | MRI-PDFF | S0: 13.9%  S1: 64.8%  S2: 12.0%  S3: 9.3% | **AUC ≥S1: 0.745, >271 dB/m**  AUC ≥S2: 0.820, >296 dB/m  AUC ≥S3: 0.836, >309 dB/m |
| Chaidez, 2022 | Colorado, US | 206  (126/80) | 13.7 (3.7) | Liver transplantation, ascites, abdominal skin disease | Liver biopsy | S0: 32.5%  S1: 8.9%  S2: 14.3%  S3: 44.3% | **Any steatosis: AUC 0.98 259 dB/m** |
| Desai, 2016 | Boston, US | 69  (43/26) | 16 (2.9) | Incomplete critical biochemical data, uninterpretable biopsy, ascites, pregnancy, implantable cardiac device | Liver biopsy | None: 66.6%  Mild/moderate: 15.9%  Severe: 17.4% | **Any steatosis: AUC 0.93 225 dB/m** |
| Jia, 2024 | Shenzhen, China | 105  (73/32) | 12.3 (3.9) | Concurrent liver disease, alcohol consumption, contra-indications for MRI | MRI-PDFF | S0: 27.6%  S1: 31.4%  S2: 21.0%  S3: 20.0% | **AUC ≥S1: 0.975, >237 dB/m**  AUC ≥S2: 0.984, >273 dB/m  AUC ≥S3: 0.997, >295 dB/m |
| Peng, 2024 | Hangzhou, China | 108  (65/43) | 11.3 (1.8) | Concurrent liver disease, alcohol consumption | MRI-PDFF | S0: 30.6%  S1: 31.5%  S2: 17.6%  S3: 20.4% | **AUC ≥S1: 0.810, >265 dB/m**  AUC ≥S2: 0.854, >299 dB/m  AUC ≥S3: 0.835, >303 dB/m |
| Runge, 2021 | Amsterdam, Netherlands | 60  (34/26) | 13.7  (12.1-16.1) | Concurrent liver disease, focal lesions in the right liver lobe, steatogenic medication, contraindications for MRI | MRI-PDFF | S0: 40%  S1: 35%  S2: 12%  S3: 13% | **AUC ≥S1: 0.795, >277 dB/m**  AUC ≥S2: 0.774, >294 dB/m  AUC ≥S3: 0.787, >349 dB/m |
| Shin, 2019 | Seoul, South Korea | 86  (62/24) | 13.1 (2.7) | Concurrent liver disease, alcohol consumption | MRI-PDFF | S0: 11.6%  S1: 29.1%  S2: 16.3%  S3: 43.0% | **AUC ≥S1: 0.941, >241 dB/m**  AUC ≥S2: 0.734, >299 dB/m  AUC ≥S3: 0.752, >303 dB/m |
| Tas, 2024 | Arkansan and Colorado, US | 82  (16/66) | 15.5 (1.4) | Subset of patients: CAP <241 or <225 dB/m  Diabetes, pregnancy, medication affecting insulin resistance | MRI-PDFF | No: 39%, mean MRI-PDFF 3.05%  Yes: 61%, mean MRI-PDFF 9.14% | **Any steatosis: AUC 0.649 281 dB/m** |
