# Supplementary material for: European Society for Pediatric Gastroenterology, Hepatology and Nutrition (ESPGHAN) steatotic liver disease special interest group position paper on screening, diagnosis and investigation of paediatric metabolic dysfunction‐associated steatotic liver disease
Source: J Pediatr Gastroenterol Nutr. 2026 Jun 30;83(3):555–76. doi: 10.1002/jpn3.70490 (PMC13539658; doi:10.1002/jpn3.70490)
Supplement: Supplementary file 3 [file JPN3-83-555-s003.docx]

| **Investigation** | **Potential diagnoses** |
| --- | --- |
| History for drugs and toxins (including alcohol and herbal remedies); urinary toxicology screen | Drug induced liver injury; alcohol-related steatotic liver disease |
| 24-hour urinary copper, serum copper, relative exchangeable copper (if available), ATP7B genetics, ophthalmological assessment for Kayser-Fleischer rings, liver biopsy for dry weight copper | Wilson disease |
| Immunoglobulin levels, antinuclear antibodies, anti-smooth muscle antibody, Anti-LKM1 (liver-kidney-microsomal 1), anti-LC1 (liver cytosol 1), anti-SLA (soluble liver antigen), anti-nuclear cytoplasmic antigen (ANCA) antibodies, faecal calprotectin, and liver biopsy | Autoimmune liver disease, inflammatory bowel disease |
| Glucose, ketones, blood gas, lactate, uric acid, total and free carnitines, acylcarnitines, urinary organic acids, muscle and/or liver biopsy | Mitochondrial disorders, fatty acid oxidation disorders, glycogen storage disorders |
| Plasma amino acids, ammonia, urinary succinylacetone | Urea cycle disorders, amino acid disorders (e.g. maple syrup urine disease, tyrosinaemia), citrin deficiency |
| Lipid profile, lysosomal acid lipase activity | Familial hyperlipidaemias, abetalipoproteinaemias, lysosomal acid lipase deficiency |
| Immunoreactive trypsin, sweat test | Cystic fibrosis |
| N-palmitoyl-O-phosphocholineserine (PPCS) | Niemann-Pick disease type C |
| Creatine kinase, muscle biopsy | Myopathies |
| Ferritin, transferrin saturation | Hereditary haemochromatosis |
| Serum bile acids, urinary bile acid profile | Bile acid synthetic disorders |
| Transferrin electrophoresis | Congenital disorders of glycosylation |
| Targeted genetic panels, whole exome/genome sequencing | Genomics England Panels:  Wilson's disease (R172)  Mitochondrial hepatopathies (R317)  Glycogen storage disorders (R274)  Congenital lipodystrophies (R158)  Familial hypercholesterolaemia (R134)  Severe, early-onset obesity (R149)  Inborn errors of metabolism (R98)  Neonatal cholestasis disorders (R171)  Microarray and Karyotype (e.g. Turner’s syndrome)  Shwachman-Diamond syndrome (R92, if anaemia and other supportive features)  See https://nhsgms-panelapp.genomicsengland.co.uk for further details of genes tests |

**Supplementary table 3. Extended investigations for patients with hepatic steatosis.** The precise extent, timing, and order of investigations will be based on the clinical context and should be determined in conjunction with relevant specialists (e.g. inherited metabolic disease specialists). The Genomics England testing panels (https://nhsgms-panelapp.genomicsengland.co.uk) are given as examples of gene panels used in the assessment of patients, given the relevant phenotype.
