# Supplementary material for: European Society for Pediatric Gastroenterology, Hepatology and Nutrition (ESPGHAN) steatotic liver disease special interest group position paper on screening, diagnosis and investigation of paediatric metabolic dysfunction‐associated steatotic liver disease
Source: J Pediatr Gastroenterol Nutr. 2026 Jun 30;83(3):555–76. doi: 10.1002/jpn3.70490 (PMC13539658; doi:10.1002/jpn3.70490)
Supplement: Supplementary file 4 [file JPN3-83-555-s004.docx]

**Supplementary table 1: results of the voting on the statements and recommendations** formulated by the

31 members of the Special Interest Group on MASLD (SIG-MASLD) contributed to 2 consecutive voting rounds. All participating members were invited to vote on agreement with statements and recommendations via Qualtrics using a four-point scale (“totally agree”; “somewhat agree”, “somewhat disagree”, “disagree”). Consensus was obtained for all statements and recommendations after the first voting round. Based on the level of agreement and the feedback provided during the first voting round, 15 statements and recommendations were reformulated and underwent a second voting round. The results are shown in resp. table 1a (first voting round) and table 1b (second voting round, statements and recommendations in bold were adapted based on the feedback from the first voting round and revoted upon).

**Table 1.a**

| **Statements and recommendation** | **Totally agree** | **Somewhat agree** | **Somewhat disagree** | **Disagree** |
| --- | --- | --- | --- | --- |
| 1. In scientific research and clinical practice, MASLD and MASH (metabolic dysfunction-associated steatohepatitis) are to be used as terminology for non-alcoholic fatty liver disease (NAFLD) | 29 (94%) | 2 (6%) |  |  |
| 2. Children ≥10 years with obesity should be screened for MASLD (LoE 4) | 21 (68%) | 7 (23%) | 3 (10%) |  |
| 3. Screen children of any age with features of metabolic syndrome (LoE 4) | 25 (81%) | 4 (13%) | 1 (3%) | 1 (3%) |
| 4. In children eligible for screening (see recommendation 2 and 3), screening for MASLD should be performed using ALT > 30 IU/L (LoE 4) | 20 (65%) | 9 (29%) | 2 (6%) |  |
| 5. Age-, sex-, pubertal stage-, and population-specific reference ranges are needed to define normal liver enzyme levels (LoE 3) | 27 (87%) | 2 (6%) | 1 (3%) | 1 (3%) |
| 6. The diagnosis of MASLD requires demonstration of steatosis on imaging or histology in an appropriate clinical setting (LoE 3) | 27 (87%) | 3 (10%) | 1 (3%) |  |
| 7. All children with suspected MASLD should have, as a minimum, first-line investigations performed (Table 1) (LoE 3) | 26 (84%) | 5 (16%) |  |  |
| 8. Further diagnostic investigations should be performed in patients who are not overweight or have any red flags (°) (LoE 4) | 30 (97%) | 1 (3%) |  |  |
| 9. Diagnosis of MASLD in non-overweight children may be established by a specialist in the presence of both steatosis and metabolic dysfunction (LoE 3) | 23 (74%) | 7 (23%) | 1 (3%) |  |
| 10. Paediatricians should assess for associated comorbidities (e.g. insulin resistance, type 2 diabetes, dyslipidaemia, arterial hypertension, kidney disease, sleep apnoea, polycystic ovary syndrome) in children with MASLD, at initial diagnosis and every 1-2 years (LoE 3) | 28 (90%) | 3 (10%) |  |  |
| 11. Refer patients with red flags (°) to specialist (LoE 3) | 30 (97%) | 1 (3%) |  |  |
| 12. In the absence of red flags, refer children with MASLD at increased risk of advanced liver disease (ALT ≥ 80 IU/L, pre-diabetes or type 2 diabetes, first-degree relative with advanced liver fibrosis) to specialists (LoE 3) | 25 (81%) | 6 (19%) |  |  |
| 13. ALT and GGT are suitable tools for identification and monitoring progression/regression of MASLD in children (LoE 2) | 19 (61%) | 8 (26%) | 4 (13%) |  |
| 14. Ultrasound B-mode of at least moderate severity (score ≥ 2 out of 3)(°) should be used to diagnose hepatic steatosis in children (LoE 3) | 24 (77%) | 4 (13%) | 3 (10%) |  |
| 15. Ultrasound should not be used to assess steatohepatitis or the degree of fibrosis in children (LoE 3) | 28 (90%) | 1 (3%) | 2 (6%) |  |
| 16. In patients with a diagnosis of MASLD, CAP may be a suitable adjunct for the quantification and monitoring of steatosis (LoE 4) | 24 (77%) | 6 (19%) |  | 1 (3%) |
| 17. CAP >280 dB/m is strongly associated with hepatic steatosis but further evidence is needed to identify optimal cut-off values for diagnosis and staging of steatosis (LoE 4) | 27 (87%) | 3 (10%) | 1 (3%) |  |
| 18. Transient elastography may be useful to exclude liver fibrosis (< 5.5 kPa) or suggest advanced chronic liver disease (> 10 kPa) in children with MASLD (LoE 3) | 23 (74%) | 6 (19%) | 2 (6%) |  |
| 19. In children with MASLD, there is insufficient evidence to accurately stage fibrosis with transient elastography liver stiffness measurements between 5.5-10 kPa (LoE 3) | 24 (77%) | 7 (23%) |  |  |
| 20. Currently, there is insufficient evidence to recommend shear-wave elastography for use in children with MASLD (LoE 4) | 23 (74%) | 7 (23%) | 1 (3%) |  |
| 21. MRI-PDFF has a high diagnostic accuracy for the quantification of hepatic lipid content in paediatric patients with MASLD and can serve as a non-invasive reference standard for the assessment and monitoring of steatosis, although it is typically not available in routine clinical practice (LoE 2) | 27 (87%) | 4 (13%) |  |  |
| 22. MRE may be useful for identification of advanced fibrosis in paediatric MASLD though validation of optimal cut-offs is needed, although it is typically not available in routine clinical practice (LoE 3) | 27 (87%) | 4 (13%) |  |  |
| 23. FIB-4, PNFI, PNFS, and APRI are not recommended for diagnosis or stratification in children with MASLD (LoE 2) | 30 (97%) | 1 (3%) |  |  |
| 24. pFIB and Fibro-PeN require further validation prior their use in clinical practice (LoE 3) | 29 (94%) | 2 (6%) |  |  |
| 25. There is insufficient evidence to recommend the use of ELF (or its components) in paediatric MASLD (LoE 3) | 27 (87%) | 4 (13%) |  |  |
| 26. Children under 5 years old with hepatic steatosis should be investigated for genetic diseases (LoE 2) | 29 (94%) | 2 (6%) |  |  |
| 27. Genetic testing (targeted panels or whole-exome) may play a role in investigating alternative causes of steatotic liver disease, particularly in lean individuals (LoE 4) | 30 (97%) | 1 (3%) |  |  |
| 28. Testing for p.Ile148Met in PNPLA3 and other common variants is currently not recommended in clinical practice (LoE 3) | 25 (81%) | 3 (10%) | 2 (6%) | 1 (3%) |
| 29. Histopathologic evaluation of MASLD in children should include a comprehensive assessment of the biopsy to discriminate between simple steatosis or steatohepatitis, including the assessment of portal inflammation and portal-based fibrosis, as well as a description of the zonal distribution of steatosis (LoE 4) | 30 (97%) | 1 (3%) |  |  |
| 30. The term 'borderline' should not be used to describe zone 1 steatohepatitis where an experienced histopathologist has identified significant portal inflammation and/or fibrosis, as this implies the presence of progressive liver disease (LoE 4) | 27 (87%) | 2 (6%) | 2 (6%) |  |
| 31. Use ultrasound-guided liver biopsy in children with MASLD, where possible (LoE 4) | 29 (94%) | 1 (3%) | 1 (3%) |  |
| 32. Liver biopsy is an accurate tool for diagnosis and follow-up/ monitoring of paediatric MASLD  (LoE 2) | 22 (71%) | 6 (19%) | 3 (10%) |  |
| 33. Liver biopsy (including repeat biopsy) should be performed in cases of diagnostic uncertainty and/or findings of more advanced liver disease (based on clinical, biochemical, imaging or transient elastography investigations) (LoE 4) | 26 (84%) | 3 (10%) |  | 2 (6%) |
| 34. Liver biopsy may be used for risk stratification in high-risk individuals to identify adolescents with fibrosing steatohepatitis to: (i) facilitate accurate follow-up under adult services, (ii) initiation of drug treatment, or (iii) for clinical trials (LoE 4) | 30 (97%) | 1 (3%) |  |  |
| 35. Each child or adolescent with MASLD needs holistic care within a multidisciplinary team for management of obesity (LoE 3) | 28 (90%) | 3 (10%) |  |  |

*Original recommendations 2 and 3 were merged

*Original statement 5 was omitted

**Table 1.b**

| **Statements and recommendation** | **Totally agree** | **Somewhat agree** | **Somewhat disagree** | **Disagree** |
| --- | --- | --- | --- | --- |
| 1. In scientific research and clinical practice, MASLD and MASH (metabolic dysfunction-associated steatohepatitis) are to be used as terminology for non-alcoholic fatty liver disease (NAFLD). | 29 (94%) | 2 (6%) |  |  |
| **2. MASLD screening should start at age 10 in children with obesity, or earlier in children with additional cardiometabolic risk factors, severe obesity, positive family history or hypopituitarism. (LoE 4)** | **28 (90%)** | **2 (6%)** | **1 (3%)** |  |
| **3. In children eligible for screening (see recommendation 2), screening for MASLD should be performed using ALT ≥ 30 IU/L. (LoE 4)** | 28 (90%) | 3 (10%) |  |  |
| 4. The diagnosis of MASLD requires demonstration of steatosis on imaging or histology in an appropriate clinical setting. (LoE 3) | 27 (87%) | 3 (10%) | 1 (3%) |  |
| 5. All children with suspected MASLD should have, as a minimum, first-line investigations performed (Table 1). (LoE 3) | 26 (84%) | 5 (16%) |  |  |
| **6. Further diagnostic investigations should be performed in patients who have any red flags (°). (LoE 4)** | **31 (100%)** |  |  |  |
| **7. Diagnosis of MASLD in non-overweight children should be established by a specialist in the presence of both steatosis and metabolic dysfunction following thorough investigation. (LoE 3)** | **28 (90%)** | **3 (10%)** |  |  |
| **8. Paediatricians should assess for associated comorbidities (e.g. insulin resistance, type 2 diabetes, dyslipidaemia, arterial hypertension, kidney disease, sleep apnoea, polycystic ovary syndrome, depression and anxiety) in children with MASLD, at initial diagnosis and during the follow-up. (LoE 3)** | **31 (100%)** |  |  |  |
| 9. Refer patients with red flags (°) to a specialist. (LoE 3) | 30 (97%) | 1 (3%) |  |  |
| **10. In the absence of red flags, refer children with MASLD at increased risk of advanced liver disease (ALT ≥ 80 IU/L, persistently abnormal ALT despite weight loss, pre-diabetes or type 2 diabetes, first-degree relative with advanced liver fibrosis) to a specialist. (LoE 3)** | **27 (87%)** | **4 (13%)** |  |  |
| **11. ALT and GGT are suitable tools for monitoring of progression/regression of MASLD in children. (LoE 2)** | **25 (81%)** | **3 (10%)** | **2 (6%)** | **1 (3%)** |
| **12. Ultrasound B-mode of at least moderate severity (score ≥ 2 out of 3)(°) can be used to diagnose hepatic steatosis in children. (LoE 3)** | **29 (94%)** | **1 (3%)** | **1 (3%)** |  |
| **13. Ultrasound is not a suitable method to assess steatohepatitis or the degree of fibrosis in children. (LoE 3)** | **31 (100%)** |  |  |  |
| **14. In patients with a diagnosis of MASLD, CAP may be a suitable adjunct for the quantification of steatosis. (LoE 4)** | **26 (84%)** | **5 (16%)** |  |  |
| **15. CAP >280 dB/m is strongly associated with hepatic steatosis but further evidence is needed to identify optimal cut-off values for diagnosis and grading of steatosis. (LoE 4)** | **29 (94%)** | **1 (3%)** | **1 (3%)** |  |
| **16. Transient elastography may be useful to exclude significant liver fibrosis ( 10 kPa) in children with MASLD. (LoE 3)** | **29 (94%)** |  | **2 (6%)** |  |
| 17. In children with MASLD, there is insufficient evidence to accurately stage fibrosis with transient elastography liver stiffness measurements between 5.5-10 kPa. (LoE 3) | 24 (77%) | 7 (23%) |  |  |
| 18. Currently, there is insufficient evidence to recommend shear-wave elastography for use in children with MASLD. (LoE 4) | 23 (74%) | 7 (23%) | 1 (3%) |  |
| 19. MRI-PDFF has a high diagnostic accuracy for the quantification of hepatic lipid content in paediatric patients with MASLD and can serve as a non-invasive reference standard for the assessment and monitoring of steatosis, although it is typically not available in routine clinical practice. (LoE 2) | 27 (87%) | 4 (13%) |  |  |
| 20. MRE may be useful for identification of advanced fibrosis in paediatric MASLD, although validation of optimal cut-offs is needed and it is typically not available in routine clinical practice. (LoE 3) | 27 (87%) | 4 (13%) |  |  |
| 21. FIB-4, PNFI, PNFS, and APRI are not recommended for diagnosis or stratification in children with MASLD. (LoE 2) | 30 (97%) | 1 (3%) |  |  |
| 22. pFIB and Fibro-PeN require further validation prior their use in clinical practice. (LoE 3) | 29 (94%) | 2 (6%) |  |  |
| 23. There is insufficient evidence to recommend the use of ELF (or its components) in paediatric MASLD. (LoE 3) | 27 (87%) | 4 (13%) |  |  |
| 24. Children under 5 years old with hepatic steatosis should be investigated for genetic diseases. (LoE 2) | 29 (94%) | 2 (6%) |  |  |
| 25. Genetic testing (targeted panels or whole-exome) may play a role in investigating alternative causes of steatotic liver disease, particularly in lean individuals. (LoE 4) | 30 (97%) | 1 (3%) |  |  |
| 26. Testing for p.Ile148Met in PNPLA3 and other common variants is currently not recommended in clinical practice. (LoE 3) | 25 (81%) | 3 (10%) | 2 (6%) | 1 (3%) |
| 27. Histopathologic evaluation of MASLD in children should include a comprehensive assessment of the biopsy to discriminate between simple steatosis or steatohepatitis, including the assessment of portal inflammation and portal-based fibrosis, as well as a description of the zonal distribution of steatosis. (LoE 4) | 30 (97%) | 1 (3%) |  |  |
| **28. Where an experienced histopathologist has identified significant portal inflammation and/or fibrosis, the term “zone 1 steatohepatitis” should be used (and not “borderline steatohepatitis”). (LoE 4)** | **29 (94%)** | **1 (3%)** |  | **1 (3%)** |
| **29.When biopsy is performed in children with MASLD, ultrasound-guided technique should be used where possible. (LoE 4)** | **29 (94%)** | **2 (6%)** |  |  |
| 30. Liver biopsy is an accurate tool for diagnosis and follow-up/ monitoring of paediatric MASLD.  (LoE 2) | 22 (71%) | 6 (19%) | 3 (10%) |  |
| 31. Liver biopsy (including repeat biopsy) should be performed in cases of diagnostic uncertainty and/or findings of more advanced liver disease (based on clinical, biochemical, imaging or transient elastography investigations). (LoE 4) | 26 (84%) | 3 (10%) |  | 2 (6%) |
| 32. Liver biopsy may be used for risk stratification in high-risk individuals to identify adolescents with fibrosing steatohepatitis to: (i) facilitate accurate follow-up under adult services, (ii) initiation of drug treatment, or (iii) for clinical trials. (LoE 4) | 30 (97%) | 1 (3%) |  |  |
| **33. Each child or adolescent with MASLD needs holistic care within a multidisciplinary team for the management of metabolic syndrome. (LoE 3)** | **29 (94%)** | **2 (6%)** |  |  |
