# Supplementary material for: European Society for Pediatric Gastroenterology, Hepatology and Nutrition (ESPGHAN) steatotic liver disease special interest group position paper on screening, diagnosis and investigation of paediatric metabolic dysfunction‐associated steatotic liver disease
Source: J Pediatr Gastroenterol Nutr. 2026 Jun 30;83(3):555–76. doi: 10.1002/jpn3.70490 (PMC13539658; doi:10.1002/jpn3.70490)
Supplement: Supplementary file 5 [file JPN3-83-555-s005.docx]

Supplementary Table 4. Studies investigating ultrasound for liver steatosis

| **Author,**  **Year** | **Location** | **Sample size (M/F)** | **Age: mean (SD) or median (IQR)** | **Exclusion criteria** | **Comparator** | **Steatosis grades** | **Results** |
| --- | --- | --- | --- | --- | --- | --- | --- |
| Draijer, 2019/  Bohte, 2011 | Amsterdam, The Netherlands | 99  (42/57) | 14.1 (2.1) | Concurrent liver disease, diabetes, parenteral feeding, steatogenic medication, contraindications for MRI | MR spectroscopy | No steatosis: 56  Steatosis: 43 | AUC 0.70 |
| El-Koofy, 2012 | Cairo, Egypt | 34  (19/15) | Range 2-13 | No NAFLD on biopsy | Liver biopsy | No steatosis: 19  Steatosis: 15 | Sensitivity 100%, specificity 68% |
| Pacifico,  2007 | Rome, Italy | 50  (34/16) | 10.5 | Concurrent liver disease, contraindications for MRI | MRI hepatic fat fraction | No steatosis: 30  Steatosis: 20 | Strong correlation between severity on US and MRI: r = 0.69; P < 0.0001 |
| Runge, 2021 | Amsterdam, The Netherlands | 60  (34/26) | 13.7  (12.1-16.1) | Concurrent liver disease, focal lesions in the right liver lobe, steatogenic medication, contraindications for MRI | MRI-PDFF | S0: 40%  S1: 35%  S2: 12%  S3: 13% | AUC ≥S1: 0.683  AUC ≥S2: 0.801  AUC ≥S3: 0.808 |
| Shannon, 2011 | Rome, Italy | 208  (132/76) | 10.8 (2.5) | Concurrent liver disease, parenteral feeding, steatogenic medication | Liver biopsy | S0: 1.0%  S1: 30.3%  S2: 41.8%  S3: 26.9% | AUC ≥S2: 0.87 |
