# Supplementary material for: European Society for Pediatric Gastroenterology, Hepatology and Nutrition (ESPGHAN) steatotic liver disease special interest group position paper on screening, diagnosis and investigation of paediatric metabolic dysfunction‐associated steatotic liver disease
Source: J Pediatr Gastroenterol Nutr. 2026 Jun 30;83(3):555–76. doi: 10.1002/jpn3.70490 (PMC13539658; doi:10.1002/jpn3.70490)
Supplement: Supplementary file 6 [file JPN3-83-555-s006.docx]

Supplementary Table 9. Studies comparing MR elastography to liver biopsy

| **Author,**  **Year** | **Location** | **Sample size (M/F)** | **Mean age (SD)** | **Device characteristics** | **Exclusion criteria** | **Fibrosis stages** | **Results** |
| --- | --- | --- | --- | --- | --- | --- | --- |
| Dilman, 2018 | Ohio, USA | 69  (44/25) | 14.3 (2.8) | 1.5T MRI  Ingenia, Philips  or HDx, GE | ≥21y, concurrent liver disease, alcohol overuse | Mean (SD) stage 1 (1.1), range 0-4 | Correlation between LSM and fibrosis ρ = 0.22, P = 0.08  No AUC given |
| Hudert, 2019 | Berlin, Germany | 50  (40/10) | 14.1 (2.1) | 1.5T MRI  Magentom Sonata, Siemens | ≥18y, concurrent liver disease, severe chronic disease, alcohol overuse, pregnancy | 30% F0  24% F1  18% F2  28% F3 | AUC ≥F1: 0.790  AUC ≥F2: 0.907  AUC ≥F3: 0.895 |
| Ravanbakhsh, 2024 | Los Angeles, US | 77 MASLD  (57/20) | 14 (12-16) | 3.0T MRI  Ingenia, Philips | Concurrent liver disease  VCTE, MRE or biopsy unavailable | 10% F0  34% F1  35% F2  19% F3  1% F4 | AUC ≥F3: 0.817 |
| Schwimmer, 2017 | California and Texas, USA | 90  (66/24) | 13.1 (2.4) | 3T MRI  Signa HDxt or Signa Excite 3.0T | Claustrophobia, weight too high, pregnancy, unreliable MR exam | 60% F0  26.7% F1  6.7% F2  5.6% F3  1.1% F4 | AUC ≥F1: 0.772  AUC ≥F3: 0.894 |
