# Supplementary material for: European Society for Pediatric Gastroenterology, Hepatology and Nutrition (ESPGHAN) steatotic liver disease special interest group position paper on screening, diagnosis and investigation of paediatric metabolic dysfunction‐associated steatotic liver disease
Source: J Pediatr Gastroenterol Nutr. 2026 Jun 30;83(3):555–76. doi: 10.1002/jpn3.70490 (PMC13539658; doi:10.1002/jpn3.70490)
Supplement: Supplementary file 7 [file JPN3-83-555-s007.docx]

| **Medical history** | |
| --- | --- |
| ***Family history*** | Cardiometabolic dysfunction, advanced liver disease, autoimmune disease,... |
| ***Previous history*** | Low birth weight, prematurity,… |
| ***Medication use*** | Medical drugs associated with weight gain or hepatic steatosis s. a. glucocorticosteroids, anticonvulsants, psychotropic medications, … |
| ***Medical symtoms*** | Abdominal pain, fatigue, neurological symptoms… |
| ***Lifestyle habits and social history*** | Diet (including sugar sweetened beverages), physical activity, screen time, low household income, food insecurity,… |
| ***Mental health*** | Including use of alcohol, smoking/vaping, illicit drugs,… |
| ***Ethnicity*** | e.g. Hispanic ethnicity |
| ***Assessment of comorbidities*** | - Obstructive sleep apnoea: snoring, apnoea, disordered sleep, morning headaches, daytime sleepiness, nocturia, enuresis,…  - Polycystic ovary syndrome: hirsutism, acne, oligomenorrhea, dysfunctional uterine bleeding |
| **Physical examination** | |
| ***Anthropometric measures to assess (central) obesity*** | Height, weight, waist and hip circumference |
| ***Signs of liver disease*** | Abdominal tenderness, hepatosplenomegaly, jaundice, … |
| ***Assessment of comorbidities*** | - Insulin resistance: acanthosis nigricans, striae rubrae,…  - Hypertension: ambulatory blood pressure measurement |
| ***Findings associated with conditions that could lead to MASLD*** | - Hypothyroidism: goiter, decreased height velocity, short stature  - Partial lipodystrophy: abnormal fat distribution  - Panhypopituitarism: papiloedema, neurological deficits, short stature, severe obesity  - Lysosomal acid lipase deficiency: xanthelasmata |

**Supplementary table 2:** Aspects of medical history and physical examination which can help in risk assessment for paediatric MASLD
