# Supplementary material for: European Society for Pediatric Gastroenterology, Hepatology and Nutrition (ESPGHAN) steatotic liver disease special interest group position paper on screening, diagnosis and investigation of paediatric metabolic dysfunction‐associated steatotic liver disease
Source: J Pediatr Gastroenterol Nutr. 2026 Jun 30;83(3):555–76. doi: 10.1002/jpn3.70490 (PMC13539658; doi:10.1002/jpn3.70490)
Supplement: Supplementary file 8 [file JPN3-83-555-s009.docx]

# Supplementary File - Recommendations

1. In scientific research and clinical practice, MASLD and MASH are to be used as terminology for non-alcoholic fatty liver disease (NAFLD) and non-alcoholic steatohepatitis, respectively. (LoE 3;100%; strong consensus)
2. MASLD screening should start at age 10 in children with obesity (BMI z-score ≥+2), or earlier in children with evidence of insulin resistance, dyslipidaemia, severe obesity (BMI z-score ≥+3), positive family history for MASLD with advanced fibrosis, or hypopituitarism. (LoE 4; 96%; strong consensus)
3. In children eligible for screening (see recommendation 2), screening for MASLD should be performed using ALT ≥30 IU/L. (LoE 4; 100%; strong consensus)
4. Diagnosis of MASLD requires demonstration of steatosis on imaging or histology (in an appropriate clinical context). (LoE 3; 97%; strong consensus)
5. All children with suspected MASLD should have, as a minimum, first-line investigations performed (Table 1). (LoE 3; 100%; strong consensus)
6. Further diagnostic investigations should be performed in patients who have any red flags (Table 2). (LoE 4; 100%; strong consensus)
7. Diagnosis of MASLD in non-overweight (BMI z-score <+1) children should be established by a specialist in the presence of both steatosis and metabolic dysfunction following thorough investigation. (LoE 3; 100%; strong consensus)
8. Paediatricians should assess for associated comorbidities (e.g. insulin resistance, type 2 diabetes, dyslipidaemia, arterial hypertension, kidney disease, sleep apnoea, polycystic ovary syndrome, depression and anxiety) in children with MASLD, at initial diagnosis and during the follow-up. (LoE 3; 100%; strong consensus)
9. Refer patients with red flags (Figure 2 and Table 2) to a specialist. (LoE 3; 100%; strong consensus)
10. In the absence of red flags, refer children with MASLD at increased risk of advanced liver disease (ALT ≥80 IU/L, persistently abnormal ALT despite weight loss, pre-diabetes or type 2 diabetes, first-degree relative with advanced liver fibrosis) to a specialist. (LoE 3; 100%; strong consensus)
11. ALT and GGT are suitable tools for identification and monitoring of progression/regression of MASLD in children. (LoE 3; 91%; consensus)
12. US B-mode of at least moderate severity (score ≥2 out of 3) can be used to diagnose hepatic steatosis in children. (LoE 3; 97%; strong consensus)
13. US is not a suitable method to assess steatohepatitis or the degree of fibrosis in children. (LoE 3; 100%; strong consensus)
14. In patients with a diagnosis of MASLD, CAP may be a suitable adjunct for the quantification of steatosis. (LoE 4; 100%; strong consensus)
15. CAP >280 dB/m is strongly associated with hepatic steatosis, but further evidence is needed to identify optimal cut-off values for diagnosis and grading of steatosis. (LoE 4; 97%; strong consensus)
16. Transient elastography may be useful to exclude significant liver fibrosis (<5.5 kPa) or suggest advanced chronic liver disease (>10 kPa) in children with MASLD. (LoE 3; 94%; strong consensus)
17. In children with MASLD, there is insufficient evidence to accurately stage fibrosis with transient elastography liver stiffness measurements between 5.5-10 kPa. (LoE 3; 100%; strong consensus)
18. Currently, there is insufficient evidence to recommend shear-wave elastography for use in children with MASLD. (LoE 4; 97%; strong consensus)
19. MRI-PDFF has a high diagnostic accuracy for the quantification of hepatic lipid content in paediatric patients with MASLD and can serve as a non-invasive reference standard for the assessment and monitoring of steatosis, although it is typically not available in routine clinical practice. (LoE 2; 100%; strong consensus)
20. MRE may be useful for identification of advanced fibrosis in paediatric MASLD, although validation of optimal cut-offs is needed, and it is typically not available in routine clinical practice. (LoE 3; 100%; strong consensus)
21. FIB-4, PNFI, PNFS, and APRI are not recommended for diagnosis or stratification in children with MASLD. (LoE 2; 100%; strong consensus)
22. pFIB and Fibro-PeN require further validation prior to their use in clinical practice. (LoE 3; 100%; strong consensus)
23. There is insufficient evidence to recommend the use of ELF (or its components) in paediatric MASLD. (LoE 3; 100%; strong consensus)
24. Children under 5 years old with hepatic steatosis should be investigated for genetic diseases. (LoE 2; 100%; strong consensus)
25. Genetic testing (targeted panels or whole-exome) may play a role in investigating alternative causes of steatotic liver disease, particularly in lean individuals. (LoE 4; 100%; strong consensus)
26. Testing for p.Ile148Met in PNPLA3 and other common variants is currently not recommended in clinical practice. (LoE 3; 91%; strong consensus)
27. Histopathologic evaluation of MASLD in children should include a comprehensive assessment of the biopsy to discriminate between simple steatosis or steatohepatitis, including the assessment of portal inflammation and portal-based fibrosis, as well as a description of the zonal distribution of steatosis. (LoE 4; 100%; strong consensus)
28. Where an experienced histopathologist has identified significant portal inflammation and/or fibrosis, the term “zone 1 steatohepatitis” should be used (and not “borderline steatohepatitis”). (LoE 4; 97%; strong consensus)
29. When biopsy is performed in children with MASLD, ultrasound-guided technique should be used where possible. (LoE 4; 100%; strong consensus)
30. Liver biopsy is an accurate tool for diagnosis and follow-up / monitoring of paediatric MASLD (LoE 2; 90%; consensus)
31. Liver biopsy (including repeat biopsy) should be performed in cases of diagnostic uncertainty and/or findings of more advanced liver disease (based on clinical, biochemical, imaging, or transient elastography investigations). (LoE 4; 94%; consensus)
32. Liver biopsy may be used for risk stratification in high-risk individuals to identify adolescents with fibrosing steatohepatitis to: (i) facilitate accurate follow-up under adult services, (ii) initiation of drug treatment, or (iii) for clinical trials. (LoE 4; 100%; strong consensus)
33. Each child or adolescent with MASLD needs holistic care within a multidisciplinary team for the management of metabolic dysfunction (LoE 3; 100%; strong consensus)
