# Supplementary material for: European Society for Pediatric Gastroenterology, Hepatology and Nutrition (ESPGHAN) steatotic liver disease special interest group position paper on screening, diagnosis and investigation of paediatric metabolic dysfunction‐associated steatotic liver disease
Source: J Pediatr Gastroenterol Nutr. 2026 Jun 30;83(3):555–76. doi: 10.1002/jpn3.70490 (PMC13539658; doi:10.1002/jpn3.70490)
Supplement: Supplementary file 9 [file JPN3-83-555-s010.docx]

Supplementary Table 5. Studies investigating quantitative ultrasound for liver steatosis

| **Author,**  **Year** | **Location** | **Sample size (M/F)** | **Age:**  **median (IQR)** | **Exclusion criteria** | **Technique** | **Comparator** | **Steatosis grades** | **AUC** |
| --- | --- | --- | --- | --- | --- | --- | --- | --- |
| Bulakci, 2023 | Istanbul, Turkey | 140  (76/64) | 15 (13-16.5) | Concurrent liver disease, MRI not feasible | Ultrasound attenuation imaging | MRI-PDFF | S0: 50.0%  S1: 22.1%  S2: 20.7%  S3: 7.1% | AUC ≥S1: 0.94  AUC ≥S2: 0.98  AUC ≥S3: 0.97 |
| Polti, 2023 | Rome, Italy | 36  (19/17) | 11 (10-14) | Concurrent liver disease, diabetes, thyroid disease | Quantitative ultrasound with automated hepatorenal index (HRI), tissue attenuation imaging (TAI) and tissue scatter distribution imaging (TSI) | MRI-PDFF | No steatosis: 14  Steatosis: 22 | AUC HRI: 0.981  AUC TAI: 0.903  AUC TSI: 0.994 |
