# Supplementary material for: European Society for Pediatric Gastroenterology, Hepatology and Nutrition (ESPGHAN) steatotic liver disease special interest group position paper on screening, diagnosis and investigation of paediatric metabolic dysfunction‐associated steatotic liver disease
Source: J Pediatr Gastroenterol Nutr. 2026 Jun 30;83(3):555–76. doi: 10.1002/jpn3.70490 (PMC13539658; doi:10.1002/jpn3.70490)
Supplement: Supplementary file 10 [file JPN3-83-555-s011.docx]

Supplementary Table 8. Studies comparing SWE for liver fibrosis to MR elastography or liver biopsy

| **Author,**  **Year** | **Location** | **Sample size (M/F)** | **Age: mean (SD) or median (IQR)** | **Exclusion criteria** | **Comparator** | **Fibrosis stages** | **AUC and cut-off** |
| --- | --- | --- | --- | --- | --- | --- | --- |
| Alhamsi, 2020 | Ohio, US | 46 (9 NASH)  (23/23) | 11.5 (8.0-14.3) | Focal liver lesion, liver disease secondary to systemic disease | Liver biopsy | 47.8% F0  10.9% F1  21.7% F2  19.6% F3 | AUC ≥F1: 0.66, cut-off >1.89m/s  AUC ≥F2: 0.75, cut-off >1.89m/s  AUC higher in subgroup without steatosis |
| Cetinic, 2022 | Gothenburg, Sweden | 33 (1 NAFLD)  (23/10) | 12.1 | IQR/median >30% | Liver biopsy | 31.3% F0  37.5% F1  21.9% F2  9.4% F3 | AUC ≥F2: 0.891, cut-off >4.7 kPa |
| Garcovich, 2017 | Rome, Italy | 69 (all NASH)  (37/32) | 12.6 (2.48) | No NASH on biopsy, SWE not feasible | Liver biopsy | 27.9% F0  48.5% F1  23.5% F2-F3 | AUC ≥F1: 0.924, cut-off >5.1 kPa  AUC ≥F2: 0.966, cut-off >6.7kPa |
| Hebelka, 2021 | Gothenburg, Sweden | 84 (2 NASH)  (50/34) | 10.2 (5.6) | Language barrier, low-quality SWE | Liver biopsy | 11.6% F0  27.9% F1  37.2% F2  14.0% F3  9.3% F4 | AUC ≥F2: 0.77, cut-off >1.24m/s |
| Lee, 2021 | Seoul, Korea | 30 (5 NAFLD)  (22/8) | 5.1 (6.1) | Low-quality SWE, different SWE device used | Liver biopsy | 43.3% F0-F1  56.7% F2-F3 | AUC ≥F2: 0.950, cut-off >11.3 kPa  AUC ≥F3: 0.924, cut-off >18.1 kPa |
| Tutar, 2014 | Istanbul, Turkey | 76 (13 NASH)  (40/36)  50 healthy controls | Median 7.7 | No liver biopsy | Liver biopsy | 22.4% F0  18.4% F1  23.7% F2  15.8% F3  19.7% F4 | AUC ≥F1: 0.963, cut-off >1.85m/s |
